# Individual differences in nonnative lexical tone perception: Effects of tone language repertoire and musical experience

**DOI:** 10.3389/fpsyg.2022.940363

**Published:** 2022-09-29

**Authors:** Xin Ru Toh, Fun Lau, Francis C. K. Wong

**Affiliations:** Division of Linguistics and Multilingual Studies, School of Humanities, Nanyang Technological University, Singapore, Singapore

**Keywords:** musical training, musical aptitude, tone language, lexical tone, tone perception, tone production, tone discrimination, tone imitation

## Abstract

This study sought to understand the effects of tone language repertoire and musical experience on nonnative lexical tone perception and production. Thirty-one participants completed a tone discrimination task, an imitation task, and a musical abilities task. Results showed that a larger tone language repertoire and musical experience both enhanced tone discrimination performance. However, the effects were not additive, as musical experience was associated with tone discrimination performance for single-tone language speakers, but such association was not seen for dual-tone language speakers. Furthermore, among single-tone language speakers, but not among dual-tone language speakers, musical experience and musical aptitude positively correlated with tone discrimination accuracy. It is thus concluded that individuals with varying extents of tone language experience may adopt different strategies when performing tone discrimination tasks; single-tone language speakers may draw on their musical expertise while dual-tone language speakers may rely on their extensive tone language experience instead.

## Introduction

Two-thirds of the languages spoken in the world are tone languages, which use pitch height and/or pitch contour in a fine-grained manner to express lexical meaning at the word level (Yip, 2002). The two main dimensions of pitch used in tone identification are pitch height and pitch direction (Gandour, 1983). In addition, lexical tones can be subdivided into level and contour tones (Abramson, 1978). A level tone remains at a relatively consistent pitch height, while a contour tone is characterised by changes in pitch height over the course of a syllable or a word. Tones are commonly transcribed with Chao tone numerals, with 1 representing the lowest pitch and 5 representing the highest pitch (Chao, 1968). The number of tones and the similarity among tones contribute to a tone system’s complexity. An example of a tone language with a relatively simple tone system is Mandarin Chinese, which has four lexical tones that are highly contrastive in pitch height and direction – Tone 1 *high-level* [55], Tone 2 *mid-rising* [35], Tone 3 *mid-dipping* [214], and Tone 4 *high-falling* [51] (Chao, 1968; Li and Thompson, 1989). On the other hand, an example of a tone language with a more complex tone system is Cantonese, which has six lexical tones of which three are level and three are contour – Tone 1 *high-level* [55], Tone 2 *high-rising* [25], Tone 3 *mid-level* [33], Tone 4 *low-falling* [21], Tone 5 *low-rising* [23], and Tone 6 *low-level* [22] (Chao, 1947; Bauer and Benedict, 1997). Compared to Mandarin Chinese, Cantonese not only has a greater number of lexical tones but also has lexical tones sharing similar acoustic features, with four out of six tones starting at approximately the same level.

### Influence of tone language experience on tone processing

Lexical tone perception has been shown to be modulated by tone language experience. Behaviourally, it has generally been established that tone language speakers outperform non-tone language speakers in native tone perception (Bent et al., 2006; Sun and Huang, 2012; Burnham et al., 2015b; Tong et al., 2015; Tsukada and Kondo, 2019; Morett, 2020) and nonnative tone perception (Lee et al., 1996; Wayland and Guion, 2004; Wayland and Li, 2008; Qin and Mok, 2013; Schaefer and Darcy, 2014, 2020; Burnham et al., 2015b). However, compared to the wealth of literature on tone perception, relatively fewer studies have examined tone production. Whereas some studies have found that tone language speakers outperform non-tone language speakers in native tone word learning (Morett, 2020), nonnative tone word learning (Cooper and Wang, 2010), and artificial or pseudoword tone language learning (Caldwell-Harris et al., 2015; Poltrock et al., 2018), Hao (2012) found that Cantonese speakers and English speakers show similar performance in producing nonnative Mandarin Chinese tones. Zhang and Peng (2017), who trained Mandarin Chinese speakers to learn Cantonese tones, found a moderate correlation between their tone perception and production but no correlation between the degree of performance change in tone perception and production, with participants showing greater improvement in tone perception than tone production after training. The influence of tone language experience on tone production as well as the relationship between tone perception and production therefore remains unclear.

Among tone language speakers, the type of tone language experience also seems to matter. Firstly, individuals who speak a greater number of tone languages may outperform those who speak fewer tone languages in tone processing. For instance, Cantonese-Mandarin Chinese speakers exhibit more robust brainstem encoding of lexical tones than Cantonese speakers (Maggu et al., 2018b), and Taiwanese-Mandarin Chinese speakers outperform Mandarin Chinese speakers in Cantonese tone identification (Wong and Lam, 2021), suggesting that speakers of two tone languages may have better tone perception than speakers of only one tone language. Wiener and Goss (2019) also found that speakers of L1 Mandarin Chinese (and L2 English) and L3 Japanese, which is a pitch accent language, outperform speakers of L1 Japanese (and L2 English) in a Japanese pitch accent discrimination task. Notably, this effect cannot be attributed to a general bilingual advantage in cognitive functions *per se*, as several studies have found no significant differences between speakers of two non-tone languages and speakers of one non-tone language in a tone discrimination task (Liu et al., 2020) and tone word learning task (Blumenfeld and Adams, 2014).

Secondly, individuals who speak a more complex tone language may outperform those who speak a simpler tone language. For example, Dong, consisting of nine tones, has a more complex tone system than Mandarin Chinese, while Lanzhou, consisting of four tones, has a simple tone system similar to Mandarin Chinese. In a study involving pure tone and harmonic tone pitch discrimination task, Dong-Mandarin Chinese speakers outperformed Lanzhou-Mandarin Chinese speakers, who performed similarly to the Mandarin Chinese speakers (Hu et al., 2020). This suggests that speakers of two tone languages may not necessarily have better pitch processing abilities than speakers of one tone language; it may be more crucial to have experience in a tone language with a more complex tone system. To date, there have been no studies explicitly examining whether individuals who speak the same number of tone languages but speak tone languages with varying complexity show different linguistic pitch processing abilities. Nonetheless, one study found that Cantonese speakers were better than English speakers at discriminating Mandarin Chinese tones, while Mandarin Chinese speakers were not better than English speakers at discriminating Cantonese tones (Lee et al., 1996), suggesting that there is some sort of hierarchy among tone languages, such that more complex tone languages equip speakers with greater tone sensitivity.

Yet, research on the tone language advantage is far from conclusive. Some studies show mixed evidence for a facilitative effect of tone language experience, which may only be observed in certain tasks and tonal contrasts (Gandour and Harshman, 1978; Francis et al., 2008; Cooper and Wang, 2010; So and Best, 2010; Chiao et al., 2011; Cooper and Wang, 2012; Hao, 2012; Tsukada et al., 2013; Wang, 2013; Tsukada, 2019). For instance, Wang (2013) found that English and Japanese speakers outperform Hmong speakers in Mandarin Chinese tone identification. Wang (2013) thus speculated that Hmong speakers may rely on pitch height rather than pitch contour when perceiving Mandarin Chinese tones, since Hmong contrasts three level tones while Mandarin Chinese does not contrast level tones. In another instance, Chiao et al. (2011) found that compared to Vietnamese listeners whose native language contains only one level tone, Taiwanese listeners whose native language contains two level tones have more difficulty discriminating level tones in a Niger-Congo language. Chiao et al. (2011) thus argued that a simpler tone system may actually be beneficial in certain non-native tone processing tasks, due to the smaller number of perceptual magnets in the native tone language. These contradictory findings highlight that whether tone language experience has a facilitative effect on tone processing is likely to be dependent on the specific tones in the source and target languages. Furthermore, some studies have found that non-tone language speakers process tones less categorically and more psychoacoustically (Hallé et al., 2004; Peng et al., 2010; Braun and Johnson, 2011; Sun and Huang, 2012; Chen et al., 2016, 2018; Liu et al., 2017; Yu et al., 2019). As such, speakers may actually benefit from having no or limited tones in their repertoire, while tone language speakers may encounter negative effects from category assimilation.

Given the widespread use of tone languages and individual variation in tone language experience, more research needs to be conducted to examine how tone language experience modulates tone processing among people of different tone language backgrounds and using tasks involving different target tone languages.

### Influence of musical experience on tone processing

Tone perception can also be influenced by other factors, most notably musical experience. There has been a vast amount of literature showing positive music-to-language transfer effects, with musicians having an advantage over nonmusicians in tone identification (Gottfried and Riester, 2000; Alexander et al., 2005; Hung and Lee, 2008; Lee and Hung, 2008; Wayland et al., 2010; Lee et al., 2014; Chang et al., 2016; Han et al., 2019), tone discrimination (Gottfried et al., 2001; Alexander et al., 2005; Delogu et al., 2006, 2010; Marie et al., 2011; Burnham et al., 2015a; Choi, 2020), tone imitation (Gottfried et al., 2001; Gottfried, 2007), and tone word learning (Wong and Perrachione, 2007).

While the literature is dense with research examining differences between ‘professional musicians’, who are typically defined in the literature as having at least 6 to 10 years of continuous formal training in Western music (Alexander et al., 2005; Wong et al., 2007; Bidelman et al., 2011; Choi, 2020), and ‘nonmusicians’ or ‘music-naïve’ individuals, who have never been formally trained, there have been very few attempts to study effects of musical experience in those who lie somewhere between the two ends of the spectrum. These may include amateur musicians and individuals who have undergone music attrition since childhood. Some studies have shown that musical training in early childhood provides sustained enhanced neural processing of speech stimuli (Skoe and Kraus, 2012; White-Schwoch et al., 2013) and improved performance in various cognitive tasks (Schellenberg, 2006; Strong and Midden, 2020) in adulthood even after musical training has ceased, suggesting that short-term musical training has long-term benefits. Yet, the research gap concerning the population in the middle of the musically trained and non-musically trained spectrum persists. Moreover, while musical training and musical aptitude are related concepts, they are distinct measurements; it is entirely possible for professional musicians to perform poorly in musicality tests, or for nonmusicians to have an innately high musical aptitude. Studies have shown that musical aptitude relates to one’s tone perception performance in both professional musicians and nonmusicians (Delogu et al., 2006, 2010; Bowles et al., 2016; Li and DeKeyser, 2017; Qin et al., 2021). Therefore, it is essential that more research be conducted with participants who have had some musical experience albeit not pursuing musicianship professionally, as well as with consideration of both years of musical training and musicality test performance as music-related variables, as such research would allow us to more fully examine the effects of musical training and musical aptitude in the general population.

### Combined influence of tone language experience and musical experience on tone processing

Although the literature indicates that either tone language experience or musical experience influences tone perception and learning, it remains inconclusive whether there may be interaction effects. Most studies comparing tone and non-tone language speakers with and without musical training have suggested that musical training confers benefits in non-tone language speakers but not in tone language speakers. Yet, several studies have obtained contradictory results.

In terms of tone perception, some studies have reported a musician advantage only in nonnative, but not native, lexical tone perception. For instance, Mok and Zuo (2012) found that for English or French speakers but not for Cantonese speakers, musicians outperform nonmusicians in Cantonese discrimination. Chen et al. (2016) as well as Liu et al. (2020) found that for Dutch monolingual or bilingual speakers but not for Mandarin Chinese speakers, musicality test scores correlated with Mandarin Chinese tone discrimination. Similarly, Chen et al. (2020) found that for English speakers but not for Mandarin Chinese speakers, musicians have stronger categorical perception of tones than nonmusicians. On the other hand, Ong et al. (2020) found that Cantonese musicians outperform Cantonese nonmusicians in the discrimination and identification of merging Cantonese tone pairs, in particular for the most difficult Tone 2/Tone 5 contrast, but perform similarly in Cantonese tone production as measured using a tone imitation task. This suggests that a musician advantage could also be present in native contrasts.

Studies on tone word learning of nonnative contrasts also yield divergent findings on whether musical experience and tone language experience have interaction effects. Several studies have found a musician advantage only among non-tone language speakers, but not in tone language speakers, indicative of an interaction effect. For example, Cooper and Wang (2012) found that for English speakers but not for Thai tone language speakers, musicians outperform nonmusicians in Cantonese tone word learning. Likewise, Laméris and Post (2022) found that for English speakers but not for Mandarin Chinese speakers, musicians outperform nonmusicians in tone categorisation and word identification in a tonal pseudolanguage. A study by Maggu et al. (2018a) further examined the effects of musical training on Thai pseudoword learning performance among English monolinguals, Mandarin Chinese monolinguals, and Cantonese-Mandarin Chinese bilinguals and found a musician advantage only in the English monolingual group. In contrast, Chan and Leung (2020) found that regardless of musical training, Cantonese speakers outperform English speakers in incidental phonological learning of artificial Thai tone-segment connections, suggesting a lack of interaction effect.

Taken together, most of the studies point towards the conclusion that the facilitative effect of musical training and tone language experience on linguistic pitch processing is not additive, yet it remains unclear how exactly the two factors interact. The discrepancy in findings may be due to the varying difficulty of stimuli and tasks. Tone processing tasks using native tone language stimuli (Mok and Zuo, 2012; Chen et al., 2016; Liu et al., 2020; Ong et al., 2020) might not be sufficiently sensitive, since the participants are presumably proficient speakers who might show ceiling effects that mask cross-domain transfer. Notably, Liu et al. (2022) found that Cantonese, Mandarin Chinese, and Thai speakers discriminate their native tones better than non-native tones. Although one study looked at tone production using an imitation task (Ong et al., 2020), other studies utilised tone word learning tasks (Cooper and Wang, 2012; Maggu et al., 2018a; Chan and Leung, 2020; Laméris and Post, 2022) which involve participants’ sensitivity to both segmental and suprasegmental features alongside a memory component linking sound to meaning, rather than tone processing specifically. Moreover, only one study has sought to examine the interaction between additional tone language experience and musical training (Maggu et al., 2018a). It is therefore challenging to draw conclusions on whether musical experience is associated with better performance in tone perception and production among individuals with varied tone language experience.

In sum, although in general tone language speakers have an advantage over non-tone language speakers in tone perception for certain tasks and tones, it is less clear whether additional tone language experience confers additional benefits. Moreover, researchers lack a nuanced understanding of how tone language experience may interact with musical experience. Therefore, our study aims to examine how variation in tone language repertoire, musical training, and musical aptitude shape one’s lexical tone perception and production. Specifically, our study aims to address these research questions:

1) Do speakers of more than one tone language outperform speakers of just one tone language in a nonnative lexical tone discrimination task, and in a tone imitation task?
2) Do the effects, if any, of tone language experience on nonnative lexical tone discrimination and imitation interact with musical experience? If so, what is the nature of this interaction?

## Materials and methods

### Participants

All participants were recruited *via* an online screening questionnaire on their handedness, language background, and music background. All participants were young adults aged between 20 and 26 (*M* = 22.90, *SD* = 1.60), had normal hearing based on an audiometric test (25 dB HL for octave frequencies from 500 Hz to 4,000 Hz), and were right-handed based on a handedness questionnaire adapted from Oldfield's (1971) Edinburgh Handedness Inventory.

A total of 34 individuals participated in this study. Three participants were excluded as they spoke three tone languages, resulting in 31 participants included in the statistical analysis. The participants varied in their tone language background and formal musical training experience. (See Supplementary Tables 1 and 2 in Appendix 1 for the language and music background of participants). All of the participants were proficient in English and Mandarin Chinese. Half of the participants spoke Mandarin Chinese as their only tone language, henceforth referred to as single-tone language speakers. The other half spoke two tone languages, with an additional tone language on top of Mandarin Chinese, henceforth referred to as dual-tone language speakers. These additional tone languages were Hokkien (*n* = 12), Teochew (*n* = 1), and Burmese (*n* = 1). The dual-tone language speakers reported an average self-rated speaking proficiency of 3.14 (SD = 1.29) and listening proficiency of 3.50 (SD = 1.09) on a 7-point Likert scale for their additional tone languages. None of the participants spoke Cantonese. In terms of musical experience, 14 participants have had no formal musical training, and 17 participants reported to have received formal musical training, with training duration ranging widely from 3 to 17 years (*M* = 9.00, *SD* = 4.62). For the dual-tone language speakers, 7 of the participants had received formal musical training and 7 have had no formal musical training. For the single-tone language speakers, 10 of the participants had received formal musical training and 7 have had no formal musical training. None of the participants were musicians by profession or had a degree in music. Therefore, our final groupings consisted of 7 dual-tone language speakers with musical training, 7 dual-tone language speakers without musical training, 10 single-tone language speakers with musical training, and 7 single-tone language speakers without musical training (Table 1).

**Table 1:** Participant demographics.

	Dual-Tone language speakers (*n* = 14)	Single-Tone language speakers (*n* = 17)
Tone languages spoken	Mandarin Chinese + Hokkien (*n* = 12) Mandarin Chinese + Teochew (*n* = 1) Mandarin Chinese + Burmese (*n* = 1)	Mandarin Chinese (*n* = 17)
Musical training received	With musical training (*n* = 7) Without musical training (*n* = 7)	With musical training (*n* = 10) Without musical training (*n* = 7)

All participants provided their written consent before participation and the procedures were approved by the Institutional Review Board at the Nanyang Technological University.

### Speech stimuli

The speech stimuli consisted of Cantonese lexical tone tokens, which were used to assess participants’ lexical tone processing. The Cantonese speech stimuli were produced by two female native Cantonese speakers who were born in Hong Kong. The recordings were done in a sound-attenuated booth. The Cantonese speech stimuli consisted of three syllables /seoi/, /jau/, and /fu/ (transcribed in Jyutping, a Cantonese romanisation system, corresponding to [sɵy], [jɐu] and [fuː] in IPA), each realised in six lexical tones. All syllables in their six lexical tones corresponded to a real word in Cantonese. (See Supplementary Table 3 in Appendix 1 for the list of Cantonese speech stimuli). To acquire more naturalistic speech, the target words were embedded within the carrier sentence, ‘下一個字係 __’ (/haa6 jat1 go3 zi6 hai6/ ‘The next word is __’), as has been done in previous studies (Liu et al., 2016; Ong et al., 2020). The target words were extracted using Audacity Team (2018), duration normalised to 750 ms, and intensity normalised to 70 dB using Praat (Boersma, 2001). This allowed us to focus solely on the parameter of pitch or fundamental frequency, an essential component of lexical and melodic units in the domains of language and music respectively, rather than any secondary acoustic cues.

### Experimental procedures

After providing their written consent, participants were seated comfortably in a sound-attenuated booth. All participants went through two experimental tasks and one supplementary task in the following order: tone discrimination task (Experimental task 1), tone imitation task (Experimental task 2), and musical abilities task (Supplementary task). Short breaks were given between tasks to prevent fatigue. The total length of time for participation was approximately 1 h, and the participants were monetarily compensated for their time upon successful completion of the experiment.

#### Experimental task 1 – Tone discrimination task

Participants were assessed on their ability to discriminate lexical tones in Cantonese, a language that was nonnative to them. An ABX discrimination task was designed and administered on a computer *via* the E-Prime 2.0 software (Psychology Software Tools, 2015) for this purpose. Before the discrimination task, each participant went through a familiarisation phase and a practice phase.

During the familiarisation phase, participants listened to the spoken tokens of the syllable /seoi/ produced by the two speakers to familiarise themselves with the voices and pitch ranges of the two speakers. The tokens were delivered *via* a headphone. There were a total of 12 distinct tokens (2 speakers × 6 tones) presented once each during this phase. Participants were explicitly informed that there were 2 speakers and were presented with all 6 tokens from one speaker before hearing the next 6 tokens from the other speaker.

In the practice phase, participants performed the ABX discrimination task with feedback. In each trial, participants listened to three spoken tokens of the syllable /jau/ delivered *via* a headphone. The first (A) and second (B) syllables were spoken by the same speaker and differed only in their lexical tones. The third (X) syllable was spoken by the other speaker and differed from either the A or B in tones. Participants were asked to judge whether the X was the same as A or B, with responses recorded *via* a keyboard press. The inter-stimulus interval was set at 1500 ms, to provide sufficient time for the processing of spoken syllables at a phonological level (Werker and Tees, 1984). Participants were asked to provide a response within 5 s. Feedback was provided at the end of each trial to indicate if the participants had answered correctly. There were 6 trials in the practice phase.

The practice phase was followed by the test phase where participants performed the ABX task without feedback, using a different syllable, /fu/. With 6 lexical tones, there were 15 possible pairwise contrasts. For each pairwise contrast, 4 ABX permutations were constructed, namely, A-B-A, A-B-B, B-A-A, and B-A-B, resulting in a total of 60 possible ABX trials. The order of presentation of these 60 trials was randomised for each participant. The inter-stimulus interval and response time limit were the same as that of the practice phase.

#### Experimental task 2 – Tone imitation task

In this task administered on a computer *via* the E-Prime 2.0 software (Psychology Software Tools, 2015), participants were asked to imitate nonnative lexical tones. The same set of speech stimuli used in Experimental Task 1 from one speaker was used. These tokens were presented to participants *via* headphones, and they were tasked to imitate each syllable as closely as possible. A practice phase using the syllable /jau/ was first introduced to familiarise participants with the task. In each trial, participants listened twice to a syllable and were asked to repeat it twice. Their speech productions were recorded using a microphone placed at an appropriate distance. There were 6 trials in the practice phase, one trial for each Cantonese lexical tone. This was followed by the test phase with 6 trials using the syllable /fu/.

The second imitation from each tone production trial in the test phase was extracted using Audacity Team (2018), duration-normalised to 750 ms, and intensity-normalised to 70 dB using Praat (Boersma, 2001) for analysis.

The participants’ tone imitation accuracy was evaluated *via* an imitated-tone identification task performed by eight native speakers of Cantonese. These evaluators were young adults aged between 18 and 27, and were born and raised in Cantonese-speaking regions such as Guangdong Province, Hong Kong, or Macau. Prior to their participation, they were screened for normal hearing (25 dB HL for octave frequencies from 500 Hz to 4,000 Hz in both ears).

The imitated-tone identification task was administered on a computer *via* the E-Prime 2.0 software (Psychology Software Tools, 2015). The evaluators were verbally briefed that they would be listening to speech stimuli produced by nonnative speakers of Cantonese, and their task was to pick the corresponding character to the speech stimuli. They were instructed to focus on the accuracy of the lexical tone instead of the pronunciation of the syllable. To discourage them from adopting an elimination strategy, they were also told not to assume that each participant produced each syllable in all six possible lexical tones.

A practice phase was introduced to familiarise evaluators with the task. In the practice phase, evaluators listened to Cantonese speech productions by a native speaker and were asked to select the corresponding Chinese character out of six possible options (representing the 6 possible tone realisations) *via* a keyboard press. There were 2 blocks in the practice phase, with the syllable /jau/ used in the first block and the syllable/fu/used in the second block. A minimum of 83.33% accuracy (at least 5 correct trials out of 6) in the second practice block was achieved before proceeding to the test phase, where the tone production tokens to be identified were those generated by our participants in the imitation task. The trials were clustered by participant, meaning that all six tokens from a single participant were presented in succession, to minimise the challenges related to intertalker variation. The presentation order of participants and tone productions within each participant was randomised.

#### Supplementary task – Musical abilities task

Participants’ musical aptitude was assessed using the Musical Ear Test (MET; Wallentin et al., 2010). There are two components to the MET: the melody subtest and the rhythm subtest. For each subtest, participants listened to 52 pairs of phrases, and had to judge whether the second phrase was the same or different (pitch violation in melody subtest or rhythmic change in rhythm subtest) compared to the first phrase. Half of the trials were ‘same’ trials and the other half were ‘different’ trials. The MET stimuli were delivered *via* headphones, and participants gave their responses on an accompanying answer sheet. All participants completed the melody subtest followed by the rhythm subtest.

### Statistical procedures

Statistical analyses were conducted using R (R Core Team, 2020). Accuracy data for the discrimination task was converted into d-prime (d’) scores. In each trial, the participant matched the target tone X with the two options (A and B) priorly given. We calculated the d’ score for each tone. For ease of explanation, we will use Tone 1 as an example to illustrate the calculation. To calculate the d’ score for Tone 1, we extracted all the trials where Tone 1 was one of the two options given, i.e., Tone 1 was either A or B in the trial. The following definitions were adopted:

1. A hit (H) was defined as the number of trials where the target tone X was Tone 1, and the target tone X was correctly identified as Tone 1.
2. A miss (M) was defined as the number of trials where the target tone X was Tone 1, and the target tone X was incorrectly identified as NOT Tone 1.
3. A false alarm (FA) was defined as the number of trials where the target tone X was NOT Tone 1 (e.g., Tone 2 or Tone 3), and the target tone X was incorrectly identified as Tone 1.
4. A correct rejection (CR) was defined as the number of trials where the target tone X was NOT Tone 1, and the target tone X was correctly identified as NOT Tone 1.

We then computed the d’ score for Tone 1 using the Psycho package (Macmillan and Creelman, 2004; Makowski, 2018) in R. Hautus adjustment was applied for extreme values. The same calculation was performed for the remaining five tones, such that in the end we obtained six d’ scores, one for each tone. Subjects’ overall mean d’ scores ranged from 0.41 to 2.47. The mean d’ scores were highly correlated with the mean discrimination accuracy scores, *r* = 0.990, *p* < 0.001, 95% CI = [0.980, 0.995].

For both the discrimination d’ scores and the imitation accuracy scores, we performed linear mixed effects analysis using the *lme4* (Bates et al., 2015) and *lmerTest* packages (Kuznetsova et al., 2017). In the first set of analyses, tone language background (single-tone vs. dual-tone language speakers) and musical training experience (with musical training vs. without musical training) and the interaction between the two were included as fixed effects. Following a significant interaction effect, *post-hoc* planned comparisons were performed to examine the effect of tone language background for each musical training group and to examine the effect of musical training experience for each tone language background group. In the second set of analyses, the categorical musical training experience factor was replaced with a continuous years of musical training factor [scaled and centered using the R function scale()]. Following a significant interaction effect, sub-group analyses were performed for each tone language background group to assess the contribution of years of music training to the dependent variable. In both sets of analyses, by-subject and by-item (Cantonese tones 1 to 6) intercepts were included as random effects. The p-value of each fixed effect was computed using the mixed() function by comparing a model constructed without the effect of interest against the full model via a likelihood ratio test. For a more comprehensive understanding of the relationship between musicality and lexical tone processing abilities, correlation analyses were also performed to examine the relationship between all music-related variables (MET melody accuracy, MET rhythm accuracy, number of years of musical training) and the dependent variable for each tone language background group. (See Supplementary material Appendix 2 for the raw data and Supplementary material Appendix 3 for the R output).

## Results

### Experimental task 1 – Tone discrimination task

**Figure 1:**
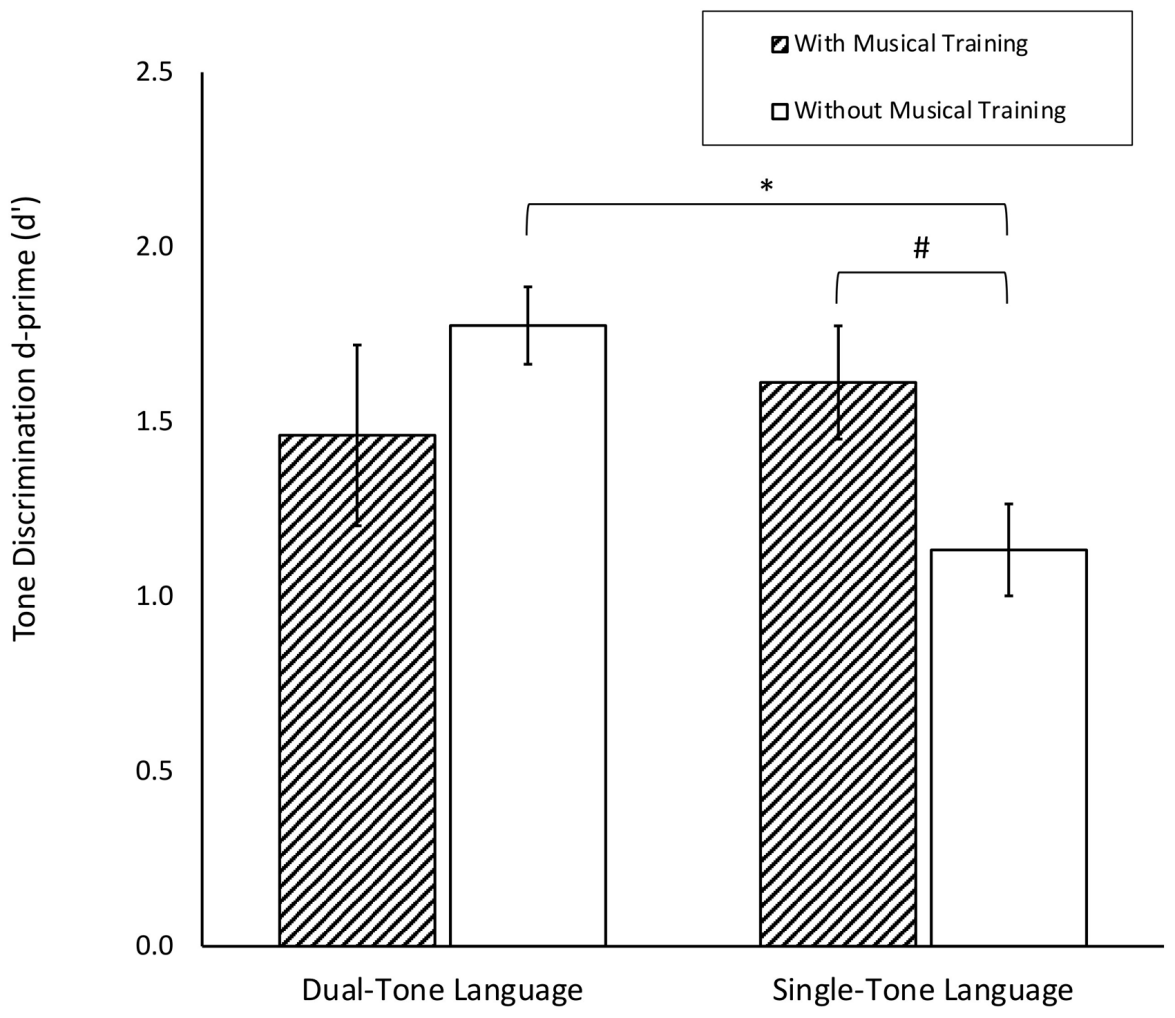
Tone discrimination d’ among single-and dual-tone language speakers with and without musical training. Error bars denote standard error. ^*^p < 0.05, ^#^*p* = 0.0563.

In the first set of analysis where musical training experience was included as a categorical factor (with musical training vs. without musical training), there was a significant effect of musical training [χ^2^(1) = 3.90, *p* < 0.05], as well as a significant interaction effect between tone language background and musical training [χ^2^(1) = 5.14, *p* < 0.05]. This interaction effect was plotted in Figure 1. *Post-hoc* comparisons with Tukey adjustment showed that the interaction was driven by two contrastive effects. Firstly, the effect of tone language background was different for the musically trained and untrained. For those without musical training, dual-tone language speakers outperformed single-tone language speakers (*p* < 0.05). For those with musical training, the single-and dual-tone language speakers performed equally well (*p* = 0.5340). Secondly, the effect of musical training experience was also different for single-and dual-tone language speakers. For dual-tone language speakers, there was an absence of musical training effect (*p* = 0.2380). For single-tone language speakers, there was a marginal effect of musical training, with the musically trained outperforming the untrained (*p* = 0.0563).

In the second set of analysis, musical training experience was included as a continuous factor of years of musical training. There was a significant effect of years of musical training [χ^2^(1) = 7.21, *p* < 0.01], as well as a significant interaction effect between tone language background and years of musical training [χ^2^(1) = 8.85, *p* < 0.01]. For single-tone language speakers, a higher number of years of musical training was associated with better tone discrimination performance (*b* = 0.2665, *SE* = 0.1060, *p* < 0.05). No such association was found for dual-tone language speakers (*b* = −0.2533, *SE* = 0.1294, *p* = 0.073).

**Figure 2:**
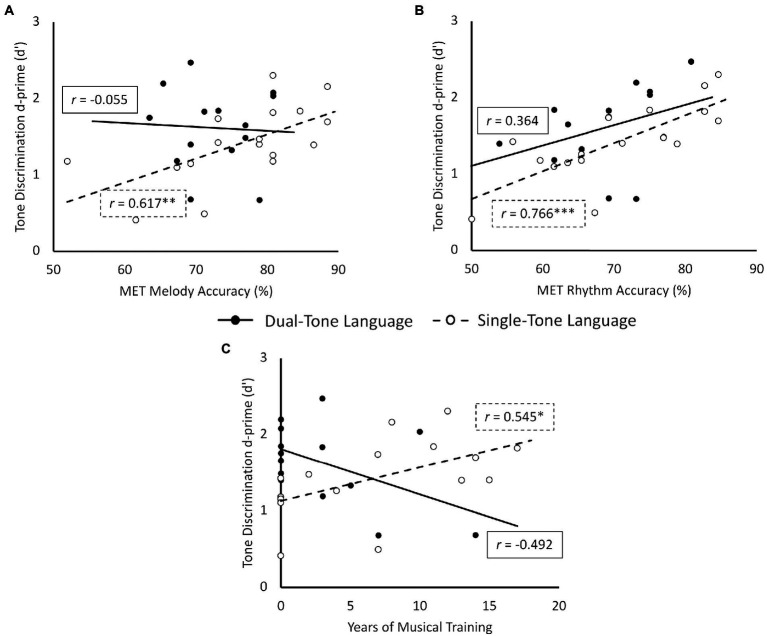
Correlation (Pearson’s *r*) between tone discrimination d’ and music-related variables: **(A)** MET Melody Accuracy, **(B)** MET Rhythm Accuracy, and **(C)** Years of Musical Training among single-and dual-tone language speakers. ^*^p < 0.05, ^**^p < 0.01, ^***^p < 0.001.

Correlation analyses with the full sample (*n* = 31) revealed a significant positive relationship between MET rhythm accuracy and d’ scores (*r* = 0.576, *p* < 0.001, 95% CI = [0.279, 0.773]). The other two music-related variables, namely MET melody accuracy and years of musical training, did not correlate (*p* > 0.05) with tone discrimination accuracy. To further explore the relationship between the three music-related factors and d’ scores in each tone language group, sub-group correlation analyses were computed. Sub-group correlation analyses were plotted in Figure 2. Among single-tone language speakers, d’ scores significantly correlated with all three factors: MET melody accuracy, *r* = 0.617, *p* < 0.01, 95% CI = [0.194, 0.847]; MET rhythm accuracy, *r* = 0.766, *p* < 0.001, 95% CI = [0.451, 0.911]; and years of musical training, *r* = 0.545, *p* = < 0.05, 95% CI = [0.087, 0.813]. There were no significant correlations among dual-tone language speakers (*r*s ranging from −0.492 to 0.364, all *p*s > 0.05).

### Experimental task 2 – Tone imitation task

Fleiss’ kappa was computed to determine if there was agreement among the eight Cantonese-speaking evaluators’ rating of the participants’ Cantonese tones imitations. There was a moderate agreement among the evaluators’ judgements, κ = 0.447, *p* < 0.001, 95% CI = [0.436, 0.459].

The participants’ tone imitation accuracy was defined as the mean percentage of tone productions that were correctly identified as the intended tone by the native evaluators. As each participant produced six tone imitations that were judged by eight native evaluators, an accuracy of 100% would require all eight evaluators to successfully identify all six tone imitations produced by the participant. Participants scored a mean of 56.86% (*SD* = 9.39%), with scores ranging from 33.33 to 70.83%.

Both sets of linear mixed effects analyses (using musical training as a categorical fixed factor and as a continuous fixed factor) resulted in singular fit, which can be indicative of an overly complex random effect structure. Therefore, we performed another round of analysis without the by-subject intercept. All analyses returned insignificant results, indicating that musical training experience, tone language background, and the interaction between the two did not have significant effects on tone imitation performance. (See Supplementary Figure 1 in Appendix 1 depicting the absence of interaction effect).

Pearson correlations were also computed to assess the relationship between tone imitation score and music-related variables, i.e., (i) MET melody accuracy, (ii) MET rhythm accuracy, and (iii) number of years of musical training. None of the correlations were significant (all *r*s < 0.128, all *p*s > 0.493).

## Discussion

This study is one of the first to examine the roles of additional tone language repertoire and musical experience in shaping one’s lexical tone perception and production. Significant findings were seen in the tone discrimination task but not in the tone imitation task.

Critically, there was a significant interaction effect between musical training and tone language background on tone discrimination d’ scores. The first key contrast driving the interaction effect was that among participants without musical training, dual-tone language speakers outperformed single-tone language speakers in tone discrimination. On the other hand, among participants with musical training, dual-tone language speakers and single-tone language speakers performed equally in tone discrimination. Previous studies comparing tone language speakers and non-tone language speakers have found that tone language experience enhances nonnative tone perception sensitivity (Lee et al., 1996; Wayland and Guion, 2004; Wayland and Li, 2008; Qin and Mok, 2013; Schaefer and Darcy, 2014; Burnham et al., 2015b; Schaefer and Darcy, 2020). Our findings further suggest that even among tone language speakers, having additional tone language experience can confer additional benefits in tone discrimination. However, the absence of tone language repertoire effects among those who have musical training suggests that the benefits conferred by tone language experience may be masked by musical training, which has also been shown to enhance tone perception (Gottfried et al., 2001; Alexander et al., 2005; Delogu et al., 2006, 2010; Marie et al., 2011; Burnham et al., 2015a; Choi, 2020). A possible explanation could be that - in line with Patel's (2011) OPERA hypothesis that explains how musical training benefits the neural encoding of speech - music requires finer pitch differentiation than lexical tone perception does, such that musical training exerts larger effects on tone perception than tone language experience does (Cooper and Wang, 2012; Patel, 2013; Ngo et al., 2016). Our findings thus suggest that there are some shared pitch processing mechanisms across language and music domains, such that either musical training or dual-tone language background enhances tone perception. Nonetheless, having a combination of both musical training and extensive tone language experience does not confer additive effects for tone processing, dovetailing with other studies in the literature (Cooper and Wang, 2012; Mok and Zuo, 2012; Maggu et al., 2018a; Chen et al., 2020; Laméris and Post, 2022).

Whereas previous studies focused on the advantage that tone language speakers have over non-tone language speakers, our study delved into the finer differentiation among tone language speakers by examining if a larger tone language repertoire confers additional benefits. In our study, among participants with no musical training, those who spoke another tone language on top of Mandarin Chinese outperformed those who only had Mandarin Chinese in their tone language repertoire. This finding can be interpreted in several ways. Firstly, as noted in the introduction, it could be that the benefits of additional tone language experience observed may be due to the number of tone languages in one’s language repertoire. Our finding converges with Maggu et al.'s (2018b) finding that Cantonese-Mandarin Chinese speakers exhibit more robust brainstem encoding of lexical tones than Cantonese speakers, as well as Wong and Lam's (2021) finding that Taiwanese-Mandarin Chinese speakers outperform Mandarin Chinese speakers in Cantonese tone identification. Individuals who speak a greater repertoire of tone languages are likely to have more extensive experience with using pitch variations at the word level for lexical distinction. In addition, individuals with a greater repertoire of tone languages are likely to have a denser tone space and more fine-grained pitch sensitivity, especially if the tone languages they speak have dissimilar tone inventories. However, this interpretation might be overly simplistic, as some studies have highlighted that apart from the amount of experience with using pitch variation at the word level, specific phonological properties in the tone systems of native languages might also affect the perception of nonnative tones (Francis et al., 2008; Cooper and Wang, 2010; So and Best, 2010; Chiao et al., 2011; Hao, 2012; Tsukada et al., 2013; Tsukada, 2019). This leads us to the second possible interpretation as mentioned in the introduction – that the benefits associated with additional tone language experience may be related to the complexity of tone languages one speaks. Our finding coheres with Hu et al.'s (2020) finding that Dong-Mandarin Chinese speakers outperformed Lanzhou-Mandarin Chinese speakers in a pure tone and harmonic tone pitch discrimination task, which they postulated was because Dong consists of nine tones while Lanzhou consists of only four tones. Likewise, most of our dual-tone language speaking participants have additional tone inventories in tone languages with more complex tone representations than Mandarin Chinese, which may have enhanced their tone processing abilities. For example, whereas Mandarin Chinese has only four tones, Hokkien – the additional tone language spoken by 12 out of 14 of our dual-tone language speakers – has up to eight tones. Specifically, Mandarin Chinese has one level tone and three highly contrastive contour tones, while Hokkien has three level tones and three contour tones alongside two stopped tones with overlapping acoustic features – Tone 1 [44] or [33], Tone 2 [24], Tone 3 [42], Tone 4 [22], Tone 5 [21], Tone 6 [22], Tone 7 [32], and Tone 8 [4(3)] (Zhou and Zhou, 2000). Mandarin Chinese speakers may attach more importance to pitch contour, whereas Hokkien speakers may be more familiar with attending to both pitch height and pitch contour as primary phonetic cues when contrasting tones. Thus, when perceiving and producing Cantonese tones that contrast in both pitch height and pitch contour, it could be the exposure to such a complex tone system that gave our dual-tone language speaking participants an edge over their counterparts who only spoke Mandarin Chinese, rather than the sheer number of tone languages spoken. In summary, our findings indicate that having a richer tone inventory can have a facilitative effect on nonnative lexical tone discrimination. However, this advantage may be dependent on individuals’ musical background, as well as the specific source and target languages (Francis et al., 2008; Cooper and Wang, 2010; So and Best, 2010; Chiao et al., 2011; Hao, 2012; Tsukada et al., 2013; Tsukada, 2019).

The second key contrast driving the interaction effect was that among single-tone language speakers, those who had received musical training marginally outperformed those without musical training in tone discrimination. Among dual-tone language speakers, the musically trained and untrained performed similarly. Moreover, when musical training was conceptualised as a continuous rather than categorical factor in our second set of analysis, we found a significant interaction effect between years of musical training and tone language background on tone discrimination accuracy. Among single-tone language speakers, a greater number of years of musical training was associated with better tone discrimination, but no such association was seen in single-tone language speakers. This indicates that musical training, especially when quantified as the years of musical training received, has differential effects on tone discrimination for persons of different tone language backgrounds. Similar observations were made from the correlation analyses, where tone discrimination accuracy was positively correlated with years of musical training as well as musical aptitude (as measured by the MET) among the single-tone language speakers, but not dual-tone language speakers. Our findings echo observations in previous studies that among non-tone language speakers but not among tone language speakers, tone processing is enhanced by musical training (Cooper and Wang, 2012; Mok and Zuo, 2012; Maggu et al., 2018a; Chen et al., 2020; Laméris and Post, 2022) or musical aptitude (Chen et al., 2016; Liu et al., 2020). Our findings further suggest that even among tone language speakers, individuals with less tone language experience benefit more greatly from musical experience for tone perception compared to individuals with additional tone language experience. Collectively, the interaction effect and subgroup correlations suggest that individuals with different extents of tone language experience use different cognitive strategies or mechanisms during lexical tone perception. Individuals with less tone language experience, including single-tone language speakers, draw on their musical expertise when performing tone discrimination tasks, such that those who have received more extensive musical training and have higher musical aptitude show better tone discrimination performance. These individuals may have a unified perception of pitch across domains and attend to acoustic differences in lexical tones in the same way that they do with musical pitch. On the other hand, individuals with richer tone language experience, such as the dual-tone language speakers in this study, may be relying on their tone language experience instead of musical expertise when performing tone discrimination tasks, explaining the lack of correlations between music-related variables and tone discrimination performance. They may perceive lexical tones as phonological categories distinct from musical pitch that does not have a phonemic role. Indeed, Chen et al. (2018) found in an ERP study on neural pitch processing that individuals lacking tone language experience seem to perceive lexical tones in a similar manner as musical melodies, whereas individuals with greater tone language experience seem to activate different neural networks when perceiving lexical tones and musical melodies. Similarly, Yu et al. (2019) found in an ERP study with second language learners of a tone language that those whose first language is not a tone language tend to process lexical tones acoustically, whereas individuals whose first language is a tone language tend to process lexical tones phonologically. Studies have found that non-tone language speakers process tones less categorically and more psychoacoustically (Hallé et al., 2004; Peng et al., 2010; Braun and Johnson, 2011; Sun and Huang, 2012; Chen et al., 2016, 2018; Liu et al., 2017). It appears that even among tone language speakers, those with less extensive tone language experience process tones more psychoacoustically and therefore benefit from musical training, while those with more extensive tone language experience process tones more phonologically and therefore do not benefit from musical training.

Among single-tone language speakers, tone discrimination scores were not only positively correlated with MET melody scores but also MET rhythm scores. Previous studies have shown that musical aptitude, and in particular melodic aptitude, is associated with tone perception performance (Delogu et al., 2006, 2010; Bowles et al., 2016; Li and DeKeyser, 2017; Qin et al., 2021). Our finding suggests that while pitch sensitivity is important in tone discrimination, temporal sensitivity is also an important cue. Distinguishing tones may not only involve paying close attention to pitch, but also involve tracking how pitch varies over time. For instance, among the six Cantonese tones used as speech stimuli in this study, Tones 2 [25], 4 [21], 5 [23] and 6 [22] share the same pitch onset in the low pitch range, but differ in their pitch movement and offset (Mok and Zuo, 2012; Qin and Mok, 2013). Indeed, temporal cues, such as the temporal encoding of fundamental frequency, amplitude envelope, and duration, contributed to tone recognition accuracy among Mandarin Chinese speakers (Kuo et al., 2008). Another study also found that Chinese children with dyslexia performed worse than age-matched controls in processing acoustic cues of speech rhythm such as rise time and intensity, as well as in Cantonese tone perception (Tong et al., 2018). These findings support recent language learning theories that general auditory sensitivity to acoustic features, including pitch and rhythm, is associated with speech perception and phonological processing skills (Tallal and Gaab, 2006; Corriveau et al., 2007; Corriveau and Goswami, 2009; Zhang and McBride-Chang, 2010).

In contrast to the tone discrimination task, no significant effect was found for the tone imitation task, and no correlations were found between tone imitation accuracy and music-related variables. Unlike naturalistic speech, tone imitation may be a relatively artificial task that mainly involves short-term acoustic memory for mimicry, and the task instructions may cue participants to speak more clearly and carefully. Furthermore, participants’ imitation tokens were evaluated by native Cantonese speakers who showed moderate agreement, which is arguably a more subjective method of evaluation compared to pitch track comparisons. However, our measure of native speaker evaluation is commonly used in the field (Liu et al., 2016; Ong et al., 2020). Our finding coheres with Ong et al.'s (2020) finding that among tone language speakers, musicians outperformed nonmusicians in tone perception but not tone production. Moreover, tone language speaking ‘tone-deaf’ amusics often show impaired tone perception but largely intact tone production (Nan et al., 2010; Liu et al., 2016). It appears that the link between tone perception and tone production might not be straightforward or robust. For instance, 3-to 6-year-old tone language speaking children are significantly better in native tone perception than production (Wong et al., 2005, 2017; Wong and Leung, 2018), and their tone perception and production accuracy show either no association (Wong et al., 2005, 2017) or weak association (Wong and Leung, 2018; Mok et al., 2019). Several studies have also investigated the tone merging phenomenon in Hong Kong, documenting a dissociative pattern in which some speakers can produce all Cantonese tones but fail to discriminate the Tone 4/Tone 6 contrast (Fung and Wong, 2010; Law et al., 2013). Collectively the findings suggest a dissociation between tone perception and production abilities, and factors contributing to tone production performance may be different from those contributing to tone perception performance.

One limitation of this study is that most of the dual-tone language speaking participants spoke Hokkien as an additional tone language, with only two participants speaking other languages instead. Therefore, the findings observed in this study could be more specific to Mandarin Chinese-Hokkien speakers and may not be generalisable to speakers of other tone languages. Future research should examine whether our findings are driven by a general effect of dual-tone language experience or by specific tone languages, possibly by recruiting participants with a wider range of tone language combinations, for instance comparing Mandarin Chinese-Hokkien vs. Mandarin Chinese-Burmese speakers.

Overall, our study indicates that tone language repertoire and musical experience interactively shape one’s tone perception abilities, as supported by the significant interaction effect of musical training and tone language background on tone discrimination accuracy as well as subgroup correlations with music-related variables. Single-tone language speakers appear to draw on their musical expertise when perceiving nonnative tones, while dual-tone language speakers appear to rely on their extensive tone language experience instead.

The findings of this study have both theoretical and pedagogical implications. Our study sheds light on whether additional tone language experience can modulate tone perception and production, which is of great relevance to researchers in the tone learning literature in terms of how they should recruit and screen participants. Moreover, it appears that although musical experience can have positive cross-domain transfer effects for linguistic pitch processing, the advantage may be more salient among non or single-tone language speakers while being masked in individuals with extensive tone language background. Although our study investigated early-stage tone processing, there may be wider implications for more complex and long-term processes like tone language learning. For instance, a learning strategy that taps on musical knowledge may be particularly beneficial for foreign tone language learners with limited tone language experience, but may be ineffective for learners with extensive tone language experience. Further research using other study designs such as training and word learning tasks can serve to deepen our understanding of whether musical experience can confer an advantage in foreign tone language learning among individuals with varying tone language repertoire.

## Data availability statement

The original contributions presented in the study are included in the Supplementary material. Further inquiries can be directed to the corresponding author.

## Ethics statement

The studies involving human participants were reviewed and approved by Nanyang Technological University. The patients/participants provided their written informed consent to participate in this study.

## Author contributions

XT and FL contributed to the conception and design of the study. XT, FL, and FW organised the database and performed the statistical analysis. XT wrote the first draft of the manuscript. XT, FL, and FW wrote sections of the manuscript. All authors contributed to the article and approved the submitted version.

## Funding

This study was supported by research grants from the Ministry of Education (MOE), Singapore (RG72/17, MOE2019-SSRTG-016 and MOE2019-T2-1-125).

## Conflict of interest

The authors declare that the research was conducted in the absence of any commercial or financial relationships that could be construed as a potential conflict of interest.

## Publisher’s note

All claims expressed in this article are solely those of the authors and do not necessarily represent those of their affiliated organisations, or those of the publisher, the editors and the reviewers. Any product that may be evaluated in this article, or claim that may be made by its manufacturer, is not guaranteed or endorsed by the publisher.

## References

[ref1] Abramson A. S. (1978). Static and dynamic acoustic cues in distinctive tones. Lang. Speech 21, 319–325. doi: 10.1177/002383097802100406, PMID: 750791

[ref2] Alexander J. A. Wong P. C. M. Bradlow A. R. (2005). Lexical tone perception in musicians and non-musicians. Paper presented at the *9th European conference on speech Communication and Technology*, Portugal.

[ref3] Audacity Team. (2018). Audacity: free audio editor and recorder (Version 2.3.2). Available at: https://www.audacityteam.org

[ref4] Bates D. Maechler M. Bolker B. Walker S. (2015). Fitting linear mixed-effects models using lme4. J. Stat. Softw. 67, 1–48. doi: 10.18637/jss.v067.i01

[ref5] Bauer R. S. Benedict P. K. (1997). Modern Cantonese phonology. New York, NY: Mouton de Greyter.

[ref6] Bent T. Bradlow A. R. Wright B. A. (2006). The influence of linguistic experience on the cognitive processing of pitch in speech and nonspeech sounds. J. Exp. Psychol. Hum. Percept. Perform. 32, 97–103. doi: 10.1037/0096-1523.32.1.97, PMID: 16478329

[ref8] Bidelman G. M. Gandour J. T. Krishnan A. (2011). Cross-domain effects of music and language experience on the representation of pitch in the human auditory brainstem. J. Cogn. Neurosci. 23, 425–434. doi: 10.1162/jocn.2009.21362, PMID: 19925180

[ref9] Blumenfeld H. K. Adams A. M. (2014). Learning and processing of nonverbal symbolic information in bilinguals and monolinguals. Front. Psychol. 5:1147. doi: 10.3389/fpsyg.2014.01147, PMID: 25360125 PMC4199272

[ref10] Boersma P. (2001). Praat, a system for doing phonetics by computer. Glot International 5, 341–345.

[ref11] Bowles A. R. Chang C. B. Karuzis V. P. (2016). Pitch ability as an aptitude for tone learning. Lang. Learn. 66, 774–808. doi: 10.1111/lang.12159

[ref12] Braun B. Johnson E. K. (2011). Question or tone 2? How language experience and linguistic function guide pitch processing. J. Phon. 39, 585–594. doi: 10.1016/j.wocn.2011.06.002

[ref13] Burnham D. Brooker R. Reid A. (2015a). The effects of absolute pitch ability and musical training on lexical tone perception. Psychol. Music 43, 881–897. doi: 10.1177/0305735614546359

[ref14] Burnham D. Kasisopa B. Reid A. Luksaneeyanawin S. Lacerda F. Virginia A. (2015b). Universality and language-specific experience in the perception of lexical tone and pitch. Appl. Psycholinguist. 36, 1459–1491. doi: 10.1017/S0142716414000496

[ref15] Caldwell-Harris C. L. Lancaster A. Ladd D. R. Dediu D. Christiansen M. H. (2015). Factors influencing sensitivity to lexical tone in an artificial language: implications for second language learning. Stud. Second. Lang. Acquis. 37, 335–357. doi: 10.1017/S0272263114000849

[ref16] Chan R. K. W. Leung J. H. C. (2020). Why are lexical tones difficult to learn? Insights from the incidental learning of tone-segment connections. Stud. Second. Lang. Acquis. 42, 33–59. doi: 10.1017/S0272263119000482

[ref17] Chang D. Hedberg N. Wang Y. (2016). Effects of musical and linguistic experience on categorization of lexical and melodic tones. J. Acoust. Soc. Am. 139, 2432–2447. doi: 10.1121/1.4947497, PMID: 27250140

[ref18] Chao Y. R. (1947). Cantonese Primer. Cambridge: Cambridge University Press.

[ref19] Chao Y. R. (1968). A grammar of spoken Chinese. Berkeley, CA: University of California Press.

[ref20] Chen A. Liu L. Kager R. (2016). Cross-domain correlation in pitch perception, the influence of native language. Language, Cognition and Neuroscience 31, 751–760. doi: 10.1080/23273798.2016.1156715

[ref21] Chen A. Peter V. Wijnen F. Schnack H. Burnham D. (2018). Are lexical tones musical? Native language’s influence on neural response to pitch in different domains. Brain Lang. 180-182, 31–41. doi: 10.1016/j.bandl.2018.04.006, PMID: 29689493

[ref22] Chen S. Zhu Y. Wayland R. Yang Y. (2020). How musical experience affects tone perception efficiency by musicians of tonal and non-tonal speakers? PLoS One 15:e0232514. doi: 10.1371/journal.pone.0232514, PMID: 32384088 PMC7209303

[ref23] Chiao W.-H. Kabak B. Braun B. (2011). When more is less: non-native perception of level tone contrasts. Proceedings of the psycholinguistic representation of tone conference, 42–45.

[ref24] Choi W. (2020). The selectivity of musical advantage: musicians exhibit perceptual advantage for some but not all Cantonese tones. Music. Percept. 37, 423–434. doi: 10.1525/MP.2020.37.5.423

[ref25] Cooper A. Wang Y. (2010). Cantonese tone word learning by tone and non-tone language speakers. Paper presented at the Interspeech, Makuhari, Chiba, Japan.

[ref26] Cooper A. Wang Y. (2012). The influence of linguistic and musical experience on Cantonese word learning. J. Acoust. Soc. Am. 131, 4756–4769. doi: 10.1121/1.4714355, PMID: 22712948

[ref27] Corriveau K. H. Goswami U. (2009). Rhythmic motor entrainment in children with speech and language impairments: tapping to the beat. Cortex: A Journal Devoted to the Study of the Nervous System and Behavior 45, 119–130. doi: 10.1016/j.cortex.2007.09.008, PMID: 19046744

[ref28] Corriveau K. H. Pasquini E. Goswami U. (2007). Basic auditory processing skills and specific language impairment: a new look at an old hypothesis. Journal of Speech Language and Hearing Research 50, 647–666. doi: 10.1044/1092-4388 17538107

[ref29] Delogu F. Lampis G. Belardinelli M. O. (2006). Music-to-language transfer effect: may melodic ability improve learning of tonal languages by native nontonal speakers? Cogn. Process. 7, 203–207. doi: 10.1007/s10339-006-0146-7, PMID: 16897065

[ref30] Delogu F. Lampis G. Belardinelli M. O. (2010). From melody to lexical tone: musical ability enhances specific aspects of foreign language perception. Eur. J. Cogn. Psychol. 22, 46–61. doi: 10.1080/09541440802708136

[ref31] Francis A. L. Ciocca V. Ma L. Fenn K. (2008). Perceptual learning of Cantonese lexical tones by tone and non-tone language speakers. J. Phon. 36, 268–294. doi: 10.1016/j.wocn.2007.06.005

[ref32] Fung R. S. Y. Wong C. S. P. (2010). Mergers and near-mergers in Hong Kong Cantonese tones. Paper presented at the tone and intonation 4, Stockholm, Sweden.

[ref33] Gandour J. T. (1983). Tone perception in far eastern languages. J. Phon. 11, 149–175. doi: 10.1016/S0095-4470(19)30813-7

[ref34] Gandour J. T. Harshman R. A. (1978). Crosslanguage differences in tone perception: a multidimensional scaling investigation. Lang. Speech 21, 1–33. doi: 10.1177/002383097802100101, PMID: 692240

[ref35] Gottfried T. L. (2007). “Music and language learning: effect of musical training on learning L2 speech contrasts” in Language experience in second language speech learning: In honor of James Emil Flege. eds. Bohn O.-S. Munro M. J., vol. 17 (Amsterdam: John Benjamins), 221–237.

[ref36] Gottfried T. L. Riester D. (2000). Relation of pitch glide perception and mandarin tone identification. J. Acoust. Soc. Am. 108:2604. doi: 10.1121/1.4743698

[ref37] Gottfried T. L. Staby A. M. Ziemer C. J. (2001). Musical experience and mandarin tone discrimination and imitation. J. Acoust. Soc. Am. 115:2545. doi: 10.1121/1.4783674

[ref38] Hallé P. A. Chang Y.-C. Best C. T. (2004). Identification and discrimination of mandarin Chinese tones by mandarin Chinese vs. French listeners. J. Phon. 32, 395–421. doi: 10.1016/S0095-4470(03)00016-0

[ref39] Han Y. Goudbeek M. Mos M. Swerts M. (2019). Mandarin tone identification by tone-naïve musicians and non-musicians in auditory-visual and auditory-only conditions. Frontiers in Communication 4, 1–14. doi: 10.3389/fcomm.2019.00070

[ref40] Hao Y.-C. (2012). Second language acquisition of mandarin Chinese tones by tonal and non-tonal language speakers. J. Phon. 40, 269–279. doi: 10.1016/j.wocn.2011.11.001

[ref210] Hu A. Wang M. Li Y. Tang Q. Gu F. (2020). Dong speakers outperform Mandarin speakers in behavioral pitch discrimination. J. Acoust. Soc. Am. 147, EL62–EL65. doi: 10.1121/10.0000604, PMID: 32006963

[ref41] Hung T.-H. Lee C.-Y. (2008). Processing linguistic and musical pitch by English-speaking musicians and non-musicians. Paper presented at the 20th north American conference on Chinese linguistics, Columbus, Ohio.

[ref42] Kuo Y.-C. Rosen S. Faulkner A. (2008). Acoustic cues to tonal contrasts in mandarin: implications for cochlear implants. J. Acoust. Soc. Am. 123, 2815–2824. doi: 10.1121/1.2896755, PMID: 18529197

[ref43] Kuznetsova A. Brockhoff P. B. Christensen R. H. B. (2017). lmerTest package: tests in linear mixed effects models. J. Stat. Softw. 82, 1–26. doi: 10.18637/jss.v082.i13

[ref44] Laméris T. J. Post B. (2022). The combined effects of L1-specific and extralinguistic factors on individual performance in a tone categorization and word identification task by English-L1 and mandarin-L1 speakers. Second. Lang. Res. 026765832210900. doi: 10.1177/02676583221090068 [Epub ahead of print]

[ref45] Law S. P. Fung R. S. Y. Kung C. (2013). An ERP study of good production Vis-à-Vis poor perception of tones in Cantonese: implications for topdown speech processing. PLoS One 8:e54396. doi: 10.1371/journal.pone.0054396, PMID: 23342146 PMC3547009

[ref46] Lee C.-Y. Hung T.-H. (2008). Identification of mandarin tones by English-speaking musicians and nonmusicians. J. Acoust. Soc. Am. 124, 3235–3248. doi: 10.1121/1.2990713, PMID: 19045807

[ref47] Lee C.-Y. Lekich A. Zhang Y. (2014). Perception of pitch height in lexical and musical tones by English-speaking musicians and nonmusicians. J. Acoust. Soc. Am. 135, 1607–1615. doi: 10.1121/1.4864473, PMID: 24606295

[ref48] Lee Y.-S. Vakoch D. A. Lee H. W. (1996). Tone perception in Cantonese and mandarin: a cross-linguistic comparison. J. Psycholinguist. Res. 25, 527–542. doi: 10.1007/BF01758181, PMID: 8865624

[ref49] Li M. DeKeyser R. (2017). Perception practice, production practice, and musical ability in L2 mandarin tone-word learning. Stud. Second. Lang. Acquis. 39, 593–620. doi: 10.1017/S0272263116000358

[ref50] Li C. N. Thompson S. A. (1989). Mandarin Chinese: A functional reference grammar. Berkeley, CA: University of California Press.

[ref51] Liu F. Chan A. H. D. Ciocca V. Roquet C. Peretz I. Wong P. C. M. (2016). Pitch perception and production in congenital amusia: evidence from Cantonese speakers. J. Acoust. Soc. Am. 140, 563–575. doi: 10.1121/1.4955182, PMID: 27475178 PMC4958102

[ref52] Liu L. Chen A. Kager R. (2017). Perception of tones in mandarin and Dutch adult listeners. Language and Linguistics 18, 622–646. doi: 10.1075/lali.18.4.03liu

[ref53] Liu L. Chen A. Kager R. (2020). Simultaneous bilinguals who do not speak a tone language show enhancement in pitch sensitivity but not in executive function. Linguistic Approaches to Bilingualism, (1879–9264) 12, 310–346. doi: 10.1075/lab.19037.liu

[ref54] Liu L. Lai R. Singh L. Kalashnikova M. Wong P. C. M. Kasisopa B. (2022). The tone atlas of perceptual discriminability and perceptual distance: four tone languages and five language groups. Brain Lang. 229:105106. doi: 10.1016/j.bandl.2022.105106, PMID: 35390675

[ref55] Macmillan N. A. Creelman C. D. (2004). Detection theory: A User’s guide (2nd Edn.). New York: Psychology Press.

[ref56] Maggu A. R. Wong P. C. M. Liu H. Wong F. C. K. (2018a). Experience-dependent influence of music and language on lexical pitch learning is not additive. Paper presented at the Interspeech, Hyderabad.

[ref57] Maggu A. R. Zong W. Law V. Wong P. C. M. (2018b). Learning two tone languages enhances the brainstem encoding of lexical tones. Paper presented at the Interspeech, Hyderabad.

[ref58] Makowski D. (2018). The psycho package: an efficient and publishing-oriented workflow for psychological science. The Journal of Open Source Software 3:470. doi: 10.21105/joss.00470

[ref59] Marie C. L. Delogu F. Lampis G. Belardinelli M. O. Besson M. (2011). Influence of musical expertise on segmental and tonal processing in mandarin Chinese. J. Cogn. Neurosci. 23, 2701–2715. doi: 10.1162/jocn.2010.21585, PMID: 20946053

[ref60] Mok P. P. K. Fung H. S. H. Li V. G. (2019). Assessing the link between perception and production in Cantonese tone acquisition. J. Speech Lang. Hear. Res. 62, 1243–1257. doi: 10.1044/2018_JSLHR-S-17-0430, PMID: 30969892

[ref61] Mok P. P. K. Zuo D. (2012). The separation between music and speech: evidence from the perception of Cantonese tones. J. Acoust. Soc. Am. 132, 2711–2720. doi: 10.1121/1.4747010, PMID: 23039463

[ref62] Morett L. M. (2020). The influence of tonal and atonal bilingualism on children’s lexical and non-lexical tone perception. Lang. Speech 63, 221–241. doi: 10.1177/0023830919834679, PMID: 30859898

[ref63] Nan Y. Sun Y. Peretz I. (2010). Congenital amusia in speakers of a tone language: association with lexical tone agnosia. Brain 133, 2635–2642. doi: 10.1093/brain/awq178 20685803

[ref64] Ngo M. K. Vu K.-P. L. Strybel T. Z. (2016). Effects of music and tonal language experience on relative pitch performance. Am. J. Psychol. 129, 125–134. doi: 10.5406/amerjpsyc.129.2.0125, PMID: 27424415

[ref65] Oldfield R. C. (1971). The assessment and analysis of handedness: the Edinburgh inventory. Neuropsychologia 9, 97–113. doi: 10.1016/0028-3932(71)90067-4, PMID: 5146491

[ref66] Ong J. H. Wong P. C. M. Liu F. (2020). Musicians show enhanced perception, but not production, of native lexical tones. J. Acoust. Soc. Am. 148, 3443–3454. doi: 10.1121/10.0002776, PMID: 33379922

[ref67] Patel A. D. (2011). Why would musical training benefit the neural encoding of speech? The OPERA hypothesis. Front. Psychol. 2:142. doi: 10.3389/fpsyg.2011.00142, PMID: 21747773 PMC3128244

[ref68] Patel A. D. (2013). “Sharing and nonsharing of brain resources for language and music” in Language, music, and the brain: A mysterious relationship. ed. Arbib M. A. (Cambridge, MA: MIT Press), 329–356.

[ref69] Peng G. Hong-Ying Z. Gong T. Yang R.-X. Kong J.-P. Wang W. S.-Y. (2010). The influence of language experience on categorical perception of pitch contours. J. Phon. 38, 616–624. doi: 10.1016/j.wocn.2010.09.003

[ref70] Poltrock S. Chen H. Kwok C. Cheung H. Nazzi T. (2018). Adult learning of novel words in a non-native language: consonants, vowels, and tones. Front. Psychol. 9, 1–15. doi: 10.3389/fpsyg.2018.01211, PMID: 30087631 PMC6066720

[ref71] Psychology Software Tools I. (2015). E-Prime 2.0 (Version 2.0.10.356). Pittsburgh, PA: Psychology Software Tools.

[ref72] Qin Z. Mok P. K. P. (2013). Discrimination of Cantonese tones by speakers of tone and non-tone languages. Kansas Linguistics 34, 26–42. doi: 10.17161/KWPL.1808.12864

[ref73] Qin Z. Zhang C. Wang W. S.-Y. (2021). The effect of mandarin listeners' musical and pitch aptitude on perceptual learning of Cantonese level-tones. J. Acoust. Soc. Am. 149, 435–446. doi: 10.1121/10.0003330, PMID: 33514138

[ref74] R Core Team. (2020). R: A language and environment for statistical computing. R Foundation for Statistical Computing, Vienna, Austria.

[ref75] Schaefer V. Darcy I. (2014). Lexical function of pitch in the first language shapes cross-linguistic perception of Thai tones. Laboratory Phonology 5, 489–522. doi: 10.1515/lp-2014-0016

[ref76] Schaefer V. Darcy I. (2020). Applying a newly learned second language dimension to the unknown: the influence of second language mandarin tones on the naïve perception of Thai tones. Psychol. Lang. Commun. 24, 90–123. doi: 10.2478/plc-2020-0007

[ref77] Schellenberg E. G. (2006). Long-term positive associations between music lessons and IQ. J. Educ. Psychol. 98, 457–468. doi: 10.1037/0022-0663.98.2.457

[ref78] Skoe E. Kraus N. (2012). A little goes a long way: how the adult brain is shaped by musical training in childhood. J. Neurosci. 32, 11507–11510. doi: 10.1523/JNEUROSCI.1949-12.2012, PMID: 22915097 PMC6703757

[ref79] So C. K. Best C. T. (2010). Cross-language perception of non-native tonal contrasts: effects of native phonological and phonetic influences. Lang. Speech 53, 273–293. doi: 10.1177/0023830909357156, PMID: 20583732 PMC2897724

[ref80] Strong J. V. Midden A. (2020). Cognitive differences between older adult instrumental musicians: benefits of continuing to play. Psychol. Music 48, 67–83. doi: 10.1177/0305735618785020

[ref81] Sun K.-C. Huang T. (2012). A cross-linguistic study of Taiwanese tone perception by Taiwanese and English listeners. J. East Asian Linguis. 21, 305–327. doi: 10.1007/s10831-012-9092-9

[ref82] Tallal P. Gaab N. (2006). Dynamic auditory processing, musical experience and language development. Trends in Neuroscience 29, 382–390. doi: 10.1016/j.tins.2006.06.003, PMID: 16806512

[ref83] Tong X. Lee S. Lee M. Burnham D. (2015). A tale of two features: perception of Cantonese lexical tone and English lexical stress in Cantonese-English bilinguals. PLoS One 10, 1–33. doi: 10.1371/journal.pone.0142896, PMID: 26606073 PMC4659673

[ref84] Tong X. Tong X. Yiu F. K. (2018). Beyond auditory sensory processing deficits: lexical tone perception deficits in Chinese children with developmental dyslexia. J. Learn. Disabil. 51, 293–301. doi: 10.1177/0022219417712018, PMID: 28608732

[ref85] Tsukada K. (2019). Are Asian language speakers similar or different? The perception of mandarin lexical tones by naïve listeners from tonal language backgrounds: a preliminary comparison of Thai and Vietnamese listeners. Australian Journal of Linguistics 39, 329–346. doi: 10.1080/07268602.2019.1620681

[ref86] Tsukada K. Kondo M. (2019). The perception of mandarin lexical tones by native speakers of Burmese. Lang. Speech 62, 625–640. doi: 10.1177/0023830918806550, PMID: 30343621

[ref87] Tsukada K. Roengpitya R. Xu H. L. Xu N. (2013). The perception of mandarin lexical tones by native Japanese and Thai listeners. J. Acoust. Soc. Am. 134:4245. doi: 10.1121/1.4831611

[ref88] Wallentin M. Nielsen A. H. Friis-Olivarius M. Vuust C. Vuust P. (2010). The musical ear test, a new reliable test for measuring musical competence. Learn. Individ. Differ. 20, 188–196. doi: 10.1016/j.lindif.2010.02.004

[ref89] Wang X. (2013). Perception of mandarin tones: the effect of L1 background and training. Mod. Lang. J. 97, 144–160. doi: 10.1111/j.1540-4781.2013.01386.x

[ref90] Wayland R. P. Guion S. G. (2004). Training English and Chinese listeners to perceive Thai tones: a preliminary report. Lang. Learn. 54, 681–712. doi: 10.1111/j.1467-9922.2004.00283.x

[ref91] Wayland R. P. Herrera E. Kaan E. (2010). Effects of musical experience and training on pitch contour perception. J. Phon. 38, 654–662. doi: 10.1016/j.wocn.2010.10.001

[ref92] Wayland R. P. Li B. (2008). Effects of two training procedures in cross-language perception of tones. J. Phon. 36, 250–267. doi: 10.1016/j.wocn.2007.06.004

[ref93] Werker J. F. Tees R. C. (1984). Phonemic and phonetic factors in adult cross-language speech perception. J. Acoust. Soc. Am. 75, 1866–1878. doi: 10.1121/1.390988, PMID: 6747097

[ref94] White-Schwoch T. Carr K. W. Anderson S. Strait D. L. Kraus N. (2013). Older adults benefit from music training early in life: biological evidence for long-term training-driven plasticity. J. Neurosci. 33, 17667–17674. doi: 10.1523/JNEUROSCI.2560-13.2013, PMID: 24198359 PMC3818545

[ref95] Wiener S. Goss S. (2019). Second and third language learners' sensitivity to Japanese pitch accent is additive: an information-based model of pitch perception. Stud. Second. Lang. Acquis. 41, 897–910. doi: 10.1017/S0272263119000068

[ref96] Wong P. Fu W. M. Cheung E. Y. L. (2017). Cantonese-speaking children do not acquire tone perception before tone production: a perceptual and acoustic study of three-year-olds' monosyllabic tones. Front. Psychol. 8, 1–15. doi: 10.3389/fpsyg.2017.01450, PMID: 28900404 PMC5581918

[ref97] Wong P. Lam K. Y. (2021). Characteristics of effective auditory training: implications from two training programs that successfully trained nonnative Cantonese tone identification in monolingual mandarin and bilingual mandarin–Taiwanese tone speakers. J. Speech Lang. Hear. Res. 64, 2490–2512. doi: 10.1044/2021_JSLHR-20-00436, PMID: 34128698

[ref98] Wong P. Leung C. T.-T. (2018). Suprasegmental features are not acquired early: perception and production of monosyllabic Cantonese lexical tones in 4-to 6-year-old preschool children. Journal of Speech Language and Hearing Research 61, 1070–1085. doi: 10.1044/2018_JSLHR-S-17-0288 29710319

[ref99] Wong P. C. M. Perrachione T. K. (2007). Learning pitch patterns in lexical identification by native English-speaking adults. Appl. Psycholinguist. 28, 565–585. doi: 10.1017/S0142716407070312

[ref100] Wong P. Schwartz R. G. Jenkins J. J. (2005). Perception and production of lexical tones by 3-year-old, mandarin-speaking children. J. Speech Lang. Hear. Res. 48, 1065–1079. doi: 10.1044/1092-4388(2005/074), PMID: 16411796

[ref101] Wong P. C. M. Skoe E. Russo N. M. Dees T. Kraus N. (2007). Musical experience shapes human brainstem encoding of linguistic pitch patterns. Nat. Neurosci. 10, 420–422. doi: 10.1038/nn1872, PMID: 17351633 PMC4508274

[ref102] Yip M. (2002). Tone. Cambridge: Cambridge University Press.

[ref103] Yu K. Li L. Chen Y. Zhou Y. Wang R. Zhang Y. (2019). Effects of native language experience on mandarin lexical tone processing in proficient second language learners. Psychophysiology 56:e13448. doi: 10.1111/psyp.13448, PMID: 31355474

[ref104] Zhang J. McBride-Chang C. (2010). Auditory sensitivity, speech perception, and reading development and impairment. Educ. Psychol. Rev. 22, 323–338. doi: 10.1007/s10648-010-9137-4

[ref105] Zhang K. Peng G. (2017). The relationship between the perception and production of non-native tones. Paper presented at the Interspeech, Stockholm, Sweden.

[ref106] Zhou C. J. Zhou Q. H. (2000). 新加坡闽南话概说 [sketch of Singapore Hokkien]. Xiamen, China: Xiamen University Publishing

