# Supplementary material for: Individual differences in nonnative lexical tone perception: Effects of tone language repertoire and musical experience
Source: Front Psychol. 2022 Sep 29;13:940363. doi: 10.3389/fpsyg.2022.940363 (PMC9557947; doi:10.3389/fpsyg.2022.940363)

Supplementary Material Appendix 1

# Supplementary Tables

**Supplementary Table 1.** Language background of participants.

| **Participant** | **Languages spoken** | **Listening proficiency** | **Speaking proficiency** |
| --- | --- | --- | --- |
| **Dual-tone language speakers with musical training** | | | |
| MTm1 | English, Mandarin Chinese, Teochew | 7, 5, 2 | 7, 5, 2 |
| MTm2 | English, Mandarin Chinese, Hokkien | 7, 5, 3 | 7, 4, 2 |
| MTm3 | English, Mandarin Chinese, Hokkien | 7, 5, 4 | 7, 5, 4 |
| MTm4 | English, Mandarin Chinese, Hokkien | 7, 2, 5 | 7, 2, 5 |
| MTm5 | English, Mandarin Chinese, Hokkien | 7, 6, 3 | 7, 6, 2 |
| MTm6 | English, Mandarin Chinese, Hokkien | 7, 4, 3 | 7, 4, 2 |
| MTm7 | English, Mandarin Chinese, Burmese | 7, 5, 6 | 7, 4, 6 |
| **Dual-tone language speakers without musical training** | | | |
| MTnm1 | English, Mandarin Chinese, Hokkien | 7, 7, 2 | 7, 7, 2 |
| MTnm2 | English, Mandarin Chinese, Hokkien | 5, 5, 4 | 5, 5, 4 |
| MTnm3 | English, Mandarin Chinese, Hokkien | 7, 5, 3 | 6, 4, 3 |
| MTnm4 | English, Mandarin Chinese, Hokkien | 6, 6, 3 | 4, 5, 2 |
| MTnm5 | English, Mandarin Chinese, Hokkien, Korean | 7, 4, 4, 1 | 7, 3, 3, 1 |
| MTnm6 | English, Mandarin Chinese, Hokkien, Korean, German | 7, 5, 4, 3, 1 | 7, 6, 4, 4, 2 |
| MTnm7 | English, Mandarin Chinese, Hokkien | 7, 6, 3 | 7, 5, 3 |
| **Single-tone language speakers with musical training** | | | |
| STm1 | English, Mandarin Chinese | 7, 5 | 7, 5 |
| STm2 | English, Mandarin Chinese | 7, 6 | 7, 6 |
| STm3 | English, Mandarin Chinese | 7, 7 | 7, 7 |
| STm4 | English, Mandarin Chinese, Japanese, Turkish | 7, 5, 4, 2 | 7, 4, 3, 1 |
| STm5 | English, Mandarin Chinese | 6, 5 | 5, 5 |
| STm6 | English, Mandarin Chinese, German, Japanese | 7, 6, 3, 2 | 7, 6, 3, 2 |
| STm7 | English, Mandarin Chinese | 7, 7 | 7, 7 |
| STm8 | English, Mandarin Chinese | 6, 4 | 6, 4 |
| STm9 | English, Mandarin Chinese | 5, 5 | 3, 1 |
| STm10 | English, Mandarin Chinese | 7, 0 | 7, 1 |
| **Single-tone language speakers without musical training** | | | |
| STnm1 | English, Mandarin Chinese | 7, 6 | 7, 4 |
| STnm2 | English, Mandarin Chinese | 7, 6 | 7, 6 |
| STnm3 | English, Mandarin Chinese | 7, 5 | 7, 5 |
| STnm4 | English, Mandarin Chinese | 5, 5 | 5, 5 |
| STnm5 | English, Mandarin Chinese | 7, 6 | 7, 6 |
| STnm6 | English, Mandarin Chinese | 7, 7 | 7, 7 |
| STnm7 | English, Mandarin Chinese | 7, 6 | 7, 5 |

**Supplementary Table 2.** Music background of participants.

| **Participant** | **Instruments learnt** | **Proficiency for each instrument** | **Age of onset for each instrument** | **Unique total number of years of training** | **Number of hours of practice each week in past 2 years** |
| --- | --- | --- | --- | --- | --- |
| **Multi tone-language speakers with musical training** | | | | | |
| MTm1 | Piano | 2 | 6 | 3 | 0 |
| MTm2 | Piano | 1 | 11 | 3 | 0 |
| MTm3 | Piano | 3 | 11 | 5 | 0 |
| MTm4 | Piano | 3 | 14 | 7 | 0 |
| MTm5 | Piano | 3 | 5 | 3 | 0 |
| MTm6 | Piano  Guitar | 6  4 | 5  13 | 14 | 0  0 |
| MTm7 | French horn  Violin  Piano | 3  4  2 | 13  17  20 | 10 | 0  5  1 |
| **Single tone-language speakers with musical training** | | | | | |
| STm1 | Piano  French horn | 3  4 | 7  13 | 12 | 0 |
| STm2 | Piano | 5 | 11 | 4 | 0 |
| STm3 | Violin  Clarinet | 5  4 | 9  13 | 7 | 0 |
| STm4 | Violin  Choir | 4  3 | 8  19 | 11 | 0  3 |
| STm5 | Violin  Piano  Viola | 2  3  2 | 9  9  13 | 8 | 0 |
| STm6 | Piano  Erhu  Violin | 5  5  5 | 7  13  19 | 13 | 1  0  0 |
| STm7 | Piano  Guitar | 5  2 | 6  18 | 15 | 6  1 |
| STm8 | Piano  Double bass | 6  4 | 4  13 | 17 | 20  0 |
| STm9 | Drums  Vocal | 6  5 | 17  20 | 7 | 4  5 |
| STm10 | Piano  Erhu  Cello  Guitar | 5  6  3  2 | 4  9  16  10 | 14 | 0.5  0.5  0  0.5 |

**Supplementary Table 3.** List of Cantonese speech stimuli.

| **Syllable in Jyutping** | **Word** | **Meaning** |
| --- | --- | --- |
| Seoi1 | 需 | Need |
| Seoi2 | 水 | Water |
| Seoi3 | 稅 | Taxes |
| Seoi4 | 誰 | Who |
| Seoi5 | 緒 | Beginning |
| Seoi6 | 睡 | Sleep |
| Jau1 | 休 | Rest |
| Jau2 | 柚 | Pomelo |
| Jau3 | 幼 | Young |
| Jau4 | 游 | Swim |
| Jau5 | 友 | Friend |
| Jau6 | 右 | Right |
| Fu1 | 夫 | Husband |
| Fu2 | 苦 | Bitter |
| Fu3 | 褲 | Trousers |
| Fu4 | 扶 | Support |
| Fu5 | 婦 | Woman |
| Fu6 | 父 | Father |

**Supplementary Figure 1.** Tone imitation accuracy among single- and dual-tone language speakers with and without musical training. Error bars denote standard error.


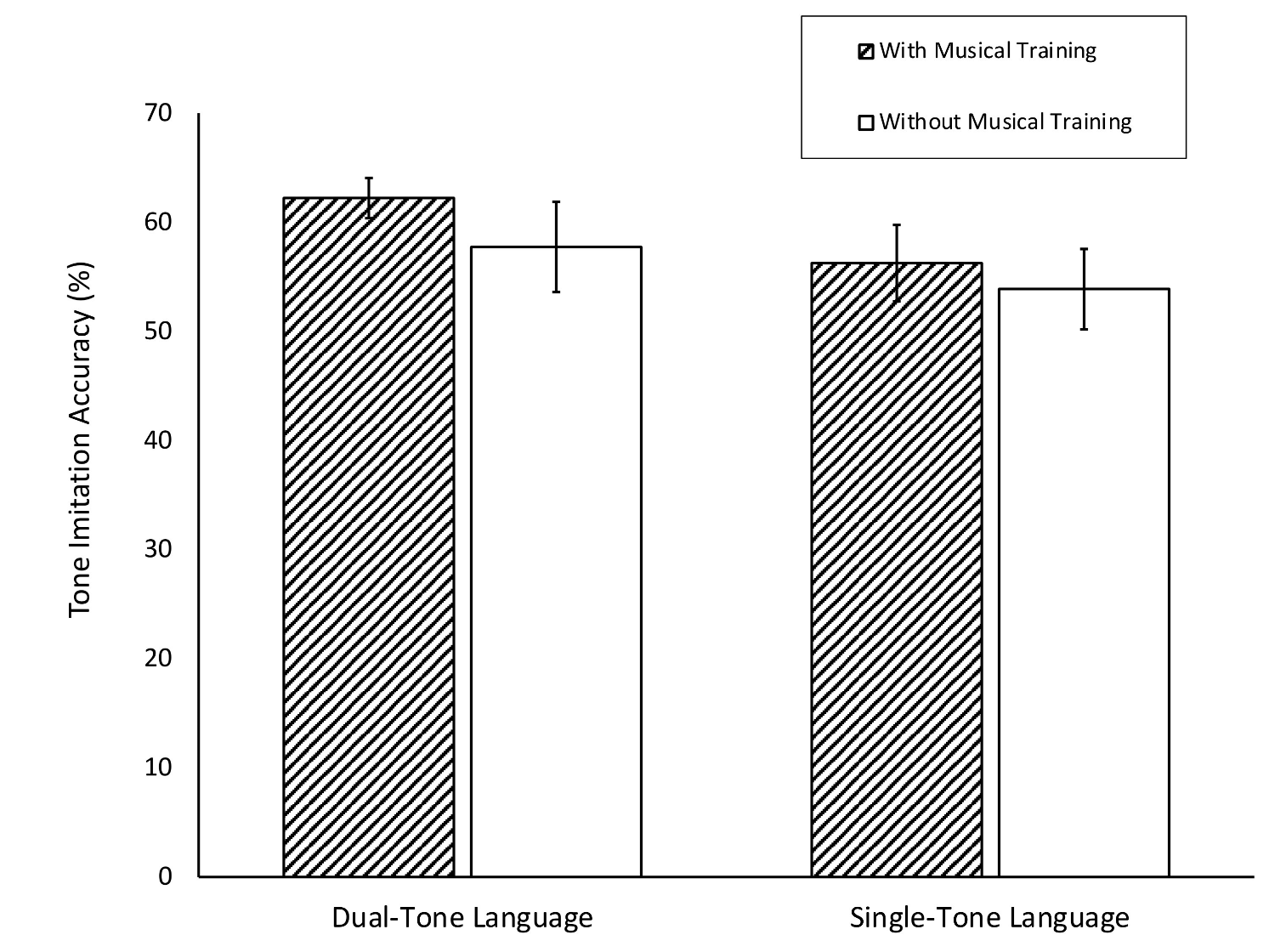

Supplement: Supplementary file 1 [file Data_Sheet_1.docx]
