# Supplementary material for: Individual differences in nonnative lexical tone perception: Effects of tone language repertoire and musical experience
Source: Front Psychol. 2022 Sep 29;13:940363. doi: 10.3389/fpsyg.2022.940363 (PMC9557947; doi:10.3389/fpsyg.2022.940363)
Supplement: Supplementary file 3 [file Data_Sheet_3.docx]

Supplementary Material Appendix 3

# R Output

> #dprime, music as categorical variable

> DISdprime.cat <- lmer(DISdprime ~ LanguageBackground * MusicBackground +(1|Subject) +(1|Tones))

> summary(DISdprime.cat)

Linear mixed model fit by REML. t-tests use Satterthwaite's method ['lmerModLmerTest']

Formula: DISdprime ~ LanguageBackground * MusicBackground + (1 | Subject) + (1 | Tones)

REML criterion at convergence: 366.5

Scaled residuals:

Min 1Q Median 3Q Max

-2.48658 -0.61023 0.01007 0.63419 2.61791

Random effects:

Groups Name Variance Std.Dev.

Subject (Intercept) 0.1888 0.4345

Tones (Intercept) 0.2508 0.5008

Residual 0.2907 0.5392

Number of obs: 186, groups: Subject, 31; Tones, 6

Fixed effects:

Estimate Std. Error df t value Pr(>|t|)

(Intercept) 3.0356 0.8524 29.7529 3.561 0.00126 **

LanguageBackground -0.9442 0.5462 27.0000 -1.729 0.09526 .

MusicBackground -1.2718 0.5462 27.0000 -2.329 0.02761 *

LanguageBackground:MusicBackground 0.7930 0.3541 27.0000 2.239 0.03358 *

---

Signif. codes: 0 ‘***’ 0.001 ‘**’ 0.01 ‘*’ 0.05 ‘.’ 0.1 ‘ ’ 1

Correlation of Fixed Effects:

(Intr) LnggBc MscBck

LnggBckgrnd -0.917

MuscBckgrnd -0.917 0.886

LnggBckg:MB 0.876 -0.946 -0.946

> mixed(DISdprime ~ MusicBackground*LanguageBackground + (1|Subject) +(1|Tones), data = lmmdata, method = "LRT")

Numerical variables NOT centered on 0: MusicBackground, LanguageBackground

If in interactions, interpretation of lower order (e.g., main) effects difficult.

REML argument to lmer() set to FALSE for method = 'PB' or 'LRT'

Fitting 4 (g)lmer() models:

[....]

Mixed Model Anova Table (Type 3 tests, LRT-method)

Model: DISdprime ~ MusicBackground * LanguageBackground + (1 | Subject) +

Model: (1 | Tones)

Data: lmmdata

Df full model: 7

Effect df Chisq p.value

1 MusicBackground 1 5.52 * .019

2 LanguageBackground 1 3.17 + .075

3 MusicBackground:LanguageBackground 1 5.14 * .023

---

Signif. codes: 0 ‘***’ 0.001 ‘**’ 0.01 ‘*’ 0.05 ‘+’ 0.1 ‘ ’ 1

> planlangxmusic = emmeans(DISdprime.cat, pairwise ~ LanguageBackground|MusicBackground, adjust = "tukey")

> planmusicxlang = emmeans(DISdprime.cat, pairwise ~ MusicBackground|LanguageBackground, adjust = "tukey")

> summary(planlangxmusic)

$emmeans

MusicBackground = 1:

LanguageBackground emmean SE df lower.CL upper.CL

1 1.61 0.256 10.8 1.048 2.18

2 1.46 0.275 13.7 0.870 2.05

MusicBackground = 2:

LanguageBackground emmean SE df lower.CL upper.CL

1 1.13 0.275 13.7 0.542 1.73

2 1.78 0.275 13.7 1.184 2.37

Degrees-of-freedom method: kenward-roger

Confidence level used: 0.95

$contrasts

MusicBackground = 1:

contrast estimate SE df t.ratio p.value

LanguageBackground1 - LanguageBackground2 0.151 0.24 27 0.630 0.5340

MusicBackground = 2:

contrast estimate SE df t.ratio p.value

LanguageBackground1 - LanguageBackground2 -0.642 0.26 27 -2.465 0.0204

Degrees-of-freedom method: kenward-roger

> summary(planmusicxlang)

$emmeans

LanguageBackground = 1:

MusicBackground emmean SE df lower.CL upper.CL

1 1.61 0.256 10.8 1.048 2.18

2 1.13 0.275 13.7 0.542 1.73

LanguageBackground = 2:

MusicBackground emmean SE df lower.CL upper.CL

1 1.46 0.275 13.7 0.870 2.05

2 1.78 0.275 13.7 1.184 2.37

Degrees-of-freedom method: kenward-roger

Confidence level used: 0.95

$contrasts

LanguageBackground = 1:

contrast estimate SE df t.ratio p.value

MusicBackground1 - MusicBackground2 0.479 0.24 27 1.995 0.0563

LanguageBackground = 2:

contrast estimate SE df t.ratio p.value

MusicBackground1 - MusicBackground2 -0.314 0.26 27 -1.207 0.2380

Degrees-of-freedom method: kenward-roger

> #dprime, music as continuous variable

> lmmdata$z_YrMusic <- scale(lmmdata$MusicYears)

> DISdprime.con <- lmer(DISdprime ~ LanguageBackground * z_YrMusic +(1|Subject) +(1|Tones))

> summary(DISdprime.con)

Linear mixed model fit by REML. t-tests use Satterthwaite's method ['lmerModLmerTest']

Formula: DISdprime ~ LanguageBackground * z_YrMusic + (1 | Subject) + (1 | Tones)

REML criterion at convergence: 365.3

Scaled residuals:

Min 1Q Median 3Q Max

-2.47127 -0.62433 0.03482 0.67675 2.59969

Random effects:

Groups Name Variance Std.Dev.

Subject (Intercept) 0.1594 0.3993

Tones (Intercept) 0.2508 0.5008

Residual 0.2907 0.5392

Number of obs: 186, groups: Subject, 31; Tones, 6

Fixed effects:

Estimate Std. Error df t value Pr(>|t|)

(Intercept) 1.1886 0.3333 22.2840 3.566 0.00170 **

LanguageBackground 0.1620 0.1745 27.0000 0.928 0.36146

z_YrMusic 0.8181 0.2587 27.0000 3.162 0.00385 **

LanguageBackground:z_YrMusic -0.5735 0.1890 27.0000 -3.034 0.00529 **

---

Signif. codes: 0 ‘***’ 0.001 ‘**’ 0.01 ‘*’ 0.05 ‘.’ 0.1 ‘ ’ 1

Correlation of Fixed Effects:

(Intr) LnggBc z_YrMs

LnggBckgrnd -0.746

z_YrMusic -0.033 -0.058

LnggBck:_YM -0.042 0.164 -0.943

> mixed(DISdprime ~ z_YrMusic*LanguageBackground + (1|Subject) +(1|Tones), data = lmmdata, method = "LRT")

Numerical variables NOT centered on 0: LanguageBackground

If in interactions, interpretation of lower order (e.g., main) effects difficult.

REML argument to lmer() set to FALSE for method = 'PB' or 'LRT'

Fitting 4 (g)lmer() models:

[....]

Mixed Model Anova Table (Type 3 tests, LRT-method)

Model: DISdprime ~ z_YrMusic * LanguageBackground + (1 | Subject) +

Model: (1 | Tones)

Data: lmmdata

Df full model: 7

Effect df Chisq p.value

1 z_YrMusic 1 9.50 ** .002

2 LanguageBackground 1 0.95 .330

3 z_YrMusic:LanguageBackground 1 8.85 ** .003

---

Signif. codes: 0 ‘***’ 0.001 ‘**’ 0.01 ‘*’ 0.05 ‘+’ 0.1 ‘ ’ 1

> detach(lmmdata)

>

> #examining effect of years of musical training on dprime in each of the two tone language background groups

> lmmdata_2tone <- lmmdata[which(lmmdata$LanguageBackground==2),]

> lmmdata_1tone <- lmmdata[which(lmmdata$LanguageBackground==1),]

> lmmdata_2tone$z_YrMusic <- scale(lmmdata_2tone$MusicYears)

> lmmdata_1tone$z_YrMusic <- scale(lmmdata_1tone$MusicYears)

> attach(lmmdata_1tone)

> DISdprime.1tone <- lmer(DISdprime ~ z_YrMusic +(1|Subject) +(1|Tones))

> summary(DISdprime.1tone)

Linear mixed model fit by REML. t-tests use Satterthwaite's method ['lmerModLmerTest']

Formula: DISdprime ~ z_YrMusic + (1 | Subject) + (1 | Tones)

REML criterion at convergence: 212.2

Scaled residuals:

Min 1Q Median 3Q Max

-2.2679 -0.6543 -0.1020 0.5928 2.5731

Random effects:

Groups Name Variance Std.Dev.

Subject (Intercept) 0.1345 0.3667

Tones (Intercept) 0.2040 0.4516

Residual 0.3271 0.5719

Number of obs: 102, groups: Subject, 17; Tones, 6

Fixed effects:

Estimate Std. Error df t value Pr(>|t|)

(Intercept) 1.4154 0.2124 7.1363 6.664 0.000264 ***

z_YrMusic 0.2665 0.1060 15.0000 2.515 0.023780 *

---

Signif. codes: 0 ‘***’ 0.001 ‘**’ 0.01 ‘*’ 0.05 ‘.’ 0.1 ‘ ’ 1

Correlation of Fixed Effects:

(Intr)

z_YrMusic 0.000

> detach(lmmdata_1tone)

> attach(lmmdata_2tone)

> DISdprime.2tone <- lmer(DISdprime ~ z_YrMusic +(1|Subject) +(1|Tones))

> summary(DISdprime.2tone)

Linear mixed model fit by REML. t-tests use Satterthwaite's method ['lmerModLmerTest']

Formula: DISdprime ~ z_YrMusic + (1 | Subject) + (1 | Tones)

REML criterion at convergence: 165.1

Scaled residuals:

Min 1Q Median 3Q Max

-1.9112 -0.6757 0.1650 0.6956 1.9666

Random effects:

Groups Name Variance Std.Dev.

Subject (Intercept) 0.1884 0.4340

Tones (Intercept) 0.2953 0.5434

Residual 0.2591 0.5090

Number of obs: 84, groups: Subject, 14; Tones, 6

Fixed effects:

Estimate Std. Error df t value Pr(>|t|)

(Intercept) 1.6184 0.2564 7.5853 6.312 0.000287 ***

z_YrMusic -0.2533 0.1294 11.9999 -1.958 0.073856 .

---

Signif. codes: 0 ‘***’ 0.001 ‘**’ 0.01 ‘*’ 0.05 ‘.’ 0.1 ‘ ’ 1

Correlation of Fixed Effects:

(Intr)

z_YrMusic 0.000

> detach(lmmdata_2tone)

> #imitation accuracy, music as categorical variable

> IMacc.cat <- lmer(IMacc ~ LanguageBackground * MusicBackground +(1|Subject) +(1|Tones), data = lmmdata)

> summary(IMacc.cat)

Linear mixed model fit by REML. t-tests use Satterthwaite's method ['lmerModLmerTest']

Formula: IMacc ~ LanguageBackground * MusicBackground + (1 | Subject) + (1 | Tones)

Data: lmmdata

REML criterion at convergence: 1692

Scaled residuals:

Min 1Q Median 3Q Max

-2.6777 -0.7594 0.1159 0.7433 2.0227

Random effects:

Groups Name Variance Std.Dev.

Subject (Intercept) 4.278 2.068

Tones (Intercept) 327.807 18.105

Residual 536.773 23.168

Number of obs: 186, groups: Subject, 31; Tones, 6

Fixed effects:

Estimate Std. Error df t value Pr(>|t|)

(Intercept) 50.5952 18.0317 31.3502 2.806 0.00855 **

LanguageBackground 8.0357 10.8556 26.9978 0.740 0.46555

MusicBackground -0.2976 10.8556 26.9978 -0.027 0.97833

LanguageBackground:MusicBackground -2.0833 7.0391 26.9978 -0.296 0.76952

---

Signif. codes: 0 ‘***’ 0.001 ‘**’ 0.01 ‘*’ 0.05 ‘.’ 0.1 ‘ ’ 1

Correlation of Fixed Effects:

(Intr) LnggBc MscBck

LnggBckgrnd -0.862

MuscBckgrnd -0.862 0.886

LnggBckg:MB 0.823 -0.946 -0.946

> mixed(IMacc ~ MusicBackground*LanguageBackground + (1|Subject) +(1|Tones), data = lmmdata, method = "LRT")

Numerical variables NOT centered on 0: MusicBackground, LanguageBackground

If in interactions, interpretation of lower order (e.g., main) effects difficult.

REML argument to lmer() set to FALSE for method = 'PB' or 'LRT'

Fitting 4 (g)lmer() models:

[boundary (singular) fit: see help('isSingular')

.boundary (singular) fit: see help('isSingular')

.boundary (singular) fit: see help('isSingular')

.boundary (singular) fit: see help('isSingular')

.]

Mixed Model Anova Table (Type 3 tests, LRT-method)

Model: IMacc ~ MusicBackground * LanguageBackground + (1 | Subject) +

Model: (1 | Tones)

Data: lmmdata

Df full model: 7

Effect df Chisq p.value

1 MusicBackground 1 0.00 .978

2 LanguageBackground 1 0.58 .447

3 MusicBackground:LanguageBackground 1 0.09 .761

---

Signif. codes: 0 ‘***’ 0.001 ‘**’ 0.01 ‘*’ 0.05 ‘+’ 0.1 ‘ ’ 1

Warning messages:

1: lme4 reported (at least) the following warnings for 'full':

* boundary (singular) fit: see help('isSingular')

2: lme4 reported (at least) the following warnings for 'MusicBackground':

* boundary (singular) fit: see help('isSingular')

3: lme4 reported (at least) the following warnings for 'LanguageBackground':

* boundary (singular) fit: see help('isSingular')

4: lme4 reported (at least) the following warnings for 'MusicBackground:LanguageBackground':

* boundary (singular) fit: see help('isSingular')

> #original model resulted in singular fit, Subject-intercept was removed.

> mixed(IMacc ~ MusicBackground*LanguageBackground + (1|Tones), data = lmmdata, method = "LRT")

Numerical variables NOT centered on 0: MusicBackground, LanguageBackground

If in interactions, interpretation of lower order (e.g., main) effects difficult.

REML argument to lmer() set to FALSE for method = 'PB' or 'LRT'

Fitting 4 (g)lmer() models:

[....]

Mixed Model Anova Table (Type 3 tests, LRT-method)

Model: IMacc ~ MusicBackground * LanguageBackground + (1 | Tones)

Data: lmmdata

Df full model: 6

Effect df Chisq p.value

1 MusicBackground 1 0.00 .978

2 LanguageBackground 1 0.58 .447

3 MusicBackground:LanguageBackground 1 0.09 .761

---

Signif. codes: 0 ‘***’ 0.001 ‘**’ 0.01 ‘*’ 0.05 ‘+’ 0.1 ‘ ’ 1

> #imitation accuracy, music as continuous variable

> IMacc.con <- lmer(IMacc ~ LanguageBackground * z_YrMusic +(1|Subject) +(1|Tones), data = lmmdata)

> summary(IMacc.con)

Linear mixed model fit by REML. t-tests use Satterthwaite's method ['lmerModLmerTest']

Formula: IMacc ~ LanguageBackground * z_YrMusic + (1 | Subject) + (1 | Tones)

Data: lmmdata

REML criterion at convergence: 1694.1

Scaled residuals:

Min 1Q Median 3Q Max

-2.6363 -0.7253 0.1630 0.7380 1.9749

Random effects:

Groups Name Variance Std.Dev.

Subject (Intercept) 3.163 1.778

Tones (Intercept) 327.779 18.105

Residual 536.769 23.168

Number of obs: 186, groups: Subject, 31; Tones, 6

Fixed effects:

Estimate Std. Error df t value Pr(>|t|)

(Intercept) 48.8198 9.2474 10.4935 5.279 0.000305 ***

LanguageBackground 5.8675 3.6825 27.0164 1.593 0.122722

z_YrMusic 2.5773 5.4617 27.0164 0.472 0.640800

LanguageBackground:z_YrMusic -0.3813 3.9901 27.0164 -0.096 0.924575

---

Signif. codes: 0 ‘***’ 0.001 ‘**’ 0.01 ‘*’ 0.05 ‘.’ 0.1 ‘ ’ 1

Correlation of Fixed Effects:

(Intr) LnggBc z_YrMs

LnggBckgrnd -0.568

z_YrMusic -0.025 -0.058

LnggBck:_YM -0.032 0.164 -0.943

> mixed(IMacc ~ z_YrMusic*LanguageBackground + (1|Subject) +(1|Tones), data = lmmdata, method = "LRT")

Numerical variables NOT centered on 0: LanguageBackground

If in interactions, interpretation of lower order (e.g., main) effects difficult.

REML argument to lmer() set to FALSE for method = 'PB' or 'LRT'

Fitting 4 (g)lmer() models:

[boundary (singular) fit: see help('isSingular')

.boundary (singular) fit: see help('isSingular')

..boundary (singular) fit: see help('isSingular')

.]

Mixed Model Anova Table (Type 3 tests, LRT-method)

Model: IMacc ~ z_YrMusic * LanguageBackground + (1 | Subject) + (1 |

Model: Tones)

Data: lmmdata

Df full model: 7

Effect df Chisq p.value

1 z_YrMusic 1 0.23 .629

2 LanguageBackground 1 2.64 .104

3 z_YrMusic:LanguageBackground 1 0.01 .922

---

Signif. codes: 0 ‘***’ 0.001 ‘**’ 0.01 ‘*’ 0.05 ‘+’ 0.1 ‘ ’ 1

Warning messages:

1: lme4 reported (at least) the following warnings for 'full':

* boundary (singular) fit: see help('isSingular')

2: lme4 reported (at least) the following warnings for 'z_YrMusic':

* boundary (singular) fit: see help('isSingular')

3: lme4 reported (at least) the following warnings for 'z_YrMusic:LanguageBackground':

* boundary (singular) fit: see help('isSingular')

> #original model resulted in singular fit, Subject-intercept was removed.

> mixed(IMacc ~ z_YrMusic*LanguageBackground + (1|Tones), data = lmmdata, method = "LRT")

Numerical variables NOT centered on 0: LanguageBackground

If in interactions, interpretation of lower order (e.g., main) effects difficult.

REML argument to lmer() set to FALSE for method = 'PB' or 'LRT'

Fitting 4 (g)lmer() models:

[....]

Mixed Model Anova Table (Type 3 tests, LRT-method)

Model: IMacc ~ z_YrMusic * LanguageBackground + (1 | Tones)

Data: lmmdata

Df full model: 6

Effect df Chisq p.value

1 z_YrMusic 1 0.23 .629

2 LanguageBackground 1 2.64 .104

3 z_YrMusic:LanguageBackground 1 0.01 .922

---

Signif. codes: 0 ‘***’ 0.001 ‘**’ 0.01 ‘*’ 0.05 ‘+’ 0.1 ‘ ’ 1
